# Single-cell transcriptomics reveals immune dysregulation mediated by IL-17A in initiation of chronic lung injuries upon real-ambient particulate matter exposure

**DOI:** 10.1186/s12989-022-00483-w

**Published:** 2022-06-23

**Authors:** Rui Zhang, Shen Chen, Liping Chen, Lizhu Ye, Yue Jiang, Hui Peng, Zhanyu Guo, Miao Li, Xinhang Jiang, Ping Guo, Dianke Yu, Rong Zhang, Yujie Niu, Yuan Zhuang, Michael Aschner, Yuxin Zheng, Daochuan Li, Wen Chen

**Affiliations:** 1 Guangdong Provincial Key Laboratory of Food, Nutrition and Health, Department of Toxicology, School of Public Health, Sun Yat-Sen University, Guangzhou, 510080 China; 2 Department of Toxicology, School of Public Health, Qingdao University, Qingdao, 266021 China; 3 Department of Toxicology, School of Public Health, Hebei Medical University, Shijiazhuang, 050017 China; 4 National Engineering Research Center of Immunological Products, Department of Microbiology and Biochemical Pharmacy, College of Pharmacy and Laboratory Medicine, Third Military Medical University, Chongqing, 400038 China; 5 Department of Molecular Pharmacology, Albert Einstein College of Medicine, Forchheimer 209, 1300 Morris Park Avenue, Bronx, NY 10461 USA

**Keywords:** Particle matter, Chronic lung injuries, Single-cell RNA sequencing, IL-17A, Myeloid-derived suppressor cells

## Abstract

**Background:**

Long-term exposure to fine particulate matter (PM_2.5_) increases susceptibility to chronic respiratory diseases, including inflammation and interstitial fibrosis. However, the regulatory mechanisms by which the immune response mediates the initiation of pulmonary fibrosis has yet to be fully characterized. This study aimed to illustrate the interplay between different cell clusters and key pathways in triggering chronic lung injuries in mice following PM exposure.

**Results:**

Six-week-old C57BL/6J male mice were exposed to PM or filtered air for 16 weeks in a real-ambient PM exposure system in Shijiazhuang, China. The transcriptional profiles of whole lung cells following sub-chronic PM exposure were characterized by analysis of single-cell transcriptomics. The IL-17A knockout (IL-17A^−/−^) mouse model was utilized to determine whether the IL-17 signaling pathway mediated immune dysregulation in PM-induced chronic lung injuries. After 16-week PM exposure, chronic lung injuries with excessive collagen deposition and increased fibroblasts, neutrophils, and monocytes were noted concurrent with a decreased number of major classes of immune cells. Single-cell analysis showed that activation of the IL-17 signaling pathway was involved in the progression of pulmonary fibrosis upon sub-chronic PM exposure. Depletion of IL-17A led to significant decline in chronic lung injuries, which was mainly triggered by reduced recruitment of myeloid-derived suppressor cells (MDSCs) and downregulation of TGF-β.

**Conclusion:**

These novel findings demonstrate that immunosuppression via the IL-17A pathway plays a critical role in the initiation of chronic lung injuries upon sub-chronic PM exposure.

**Supplementary Information:**

The online version contains supplementary material available at 10.1186/s12989-022-00483-w.

## Background

Exposure to fine particulate matter (PM_2.5_) has been invoked in the development of a variety of human diseases [1]. Particularly, increased susceptibility to chronic respiratory diseases, such as chronic obstructive pulmonary disease (COPD), pulmonary fibrosis and lung cancer has been linked to PM exposure [2, 3]. Inherent to PM-induced acute inflammation is the activation of inflammatory cells such as neutrophils and lymphocytes, and their infiltration into the lung tissue, where they interact with stromal cells to release cytokines, generating a microenvironment that triggers and favors chronic inflammation [4]. Previously, we installed a real-ambient PM exposure system in Shijiazhuang city, which is located 270 km from Beijing with the annual mean concentration of PM2.5 ranking among the top five cities with the highest PM2.5 concentration in China [5, 6]. With this device, we conducted sub-chronic real-ambient PM exposure in C57BL/6J male mice and observed chronic lung injuries and multi-organ injuries following 12-week PM exposure [5].

Persistent chronic inflammation is considered to be one of the most important initiators of progressive pulmonary fibrosis [7]. The pathological features of pulmonary fibrosis are characterized by enhanced proliferation of fibroblasts, activation of fibroblasts, deposit of extracellular matrix, and irreversible destruction of the lung architecture. The lung is a complex organ containing both resident and recruited immune cells, as well stromal cells which show differential susceptibility to various environmental stimulus [8]. Strong evidence indicates that abnormal lung epithelium expresses numerous mediators that can lead to mesenchymal-cell activation and lung remodeling in response to PM-induced pulmonary fibrosis [9]. The key molecular events involved in this process include activation of transforming growth factor-β (TGF-β), expression of multiple matrix molecules, epithelial-mesenchymal-transition (EMT), and activated profibrotic-signaling pathways [10]. Although perturbation of pulmonary immune functions plays a crucial role in mediating chronic inflammation [11], the interactions between immune cells and stromal cells and the ensuing activated inflammatory cascades in response to PM exposure, which led to initiation and progression of pulmonary fibrosis have yet to be fully characterized.

Previous studies have shown that persistent oxidative stress, inflammatory injury, and impaired immune responses contribute to PM exposure-induced chronic respiratory diseases [12]. However, it remains unclear what triggers the transition from acute inflammation to pulmonary fibrosis and which key signaling pathways are involved in the regulation of these pathophysiologic processes. The rapid development of single-cell RNA sequencing (scRNA-seq) technology permits us to identify the most sensitive cells and key regulators as well their interactions in contributing to the progression of pulmonary fibrosis [13, 14]. A comprehensive single-cell profile of whole lung tissues at the initiation stage of pulmonary fibrosis is essential for addressing how immunopathological alterations mediate PM-induced chronic lung injuries.

In this study, we performed scRNA-seq on whole lung cells from C57BL/6J male mice continuously exposed to PM for 4 months. Integrating the computational analysis with the histopathological changes, we confirmed the emergence of interstitial fibrosis characterized by chronic inflammation, excessive collagen deposition, and immune dysfunctions. Notably, activation of IL-17 signaling pathway displayed distinct patterns in cell clusters of the PM-exposed group, implicating its role in triggering chronic inflammation. In addition, we demonstrated that knocking out of IL-17A greatly attenuated the PM-induced chronic inflammation. Moreover, IL-17A participated in recruiting the myeloid-derived suppressor cells (MDSCs) and directly regulated TGF-β at both the mRNA and the protein levels. These findings provide novel insights into PM exposure-associated molecular and cellular alterations, and the regulatory mechanism of immune functions on the progression of PM-induced pulmonary fibrosis.

## Results

### Sub-chronic real-ambient PM exposure induces chronic lung injuries in C57BL/6J mice

**Fig. 1:**
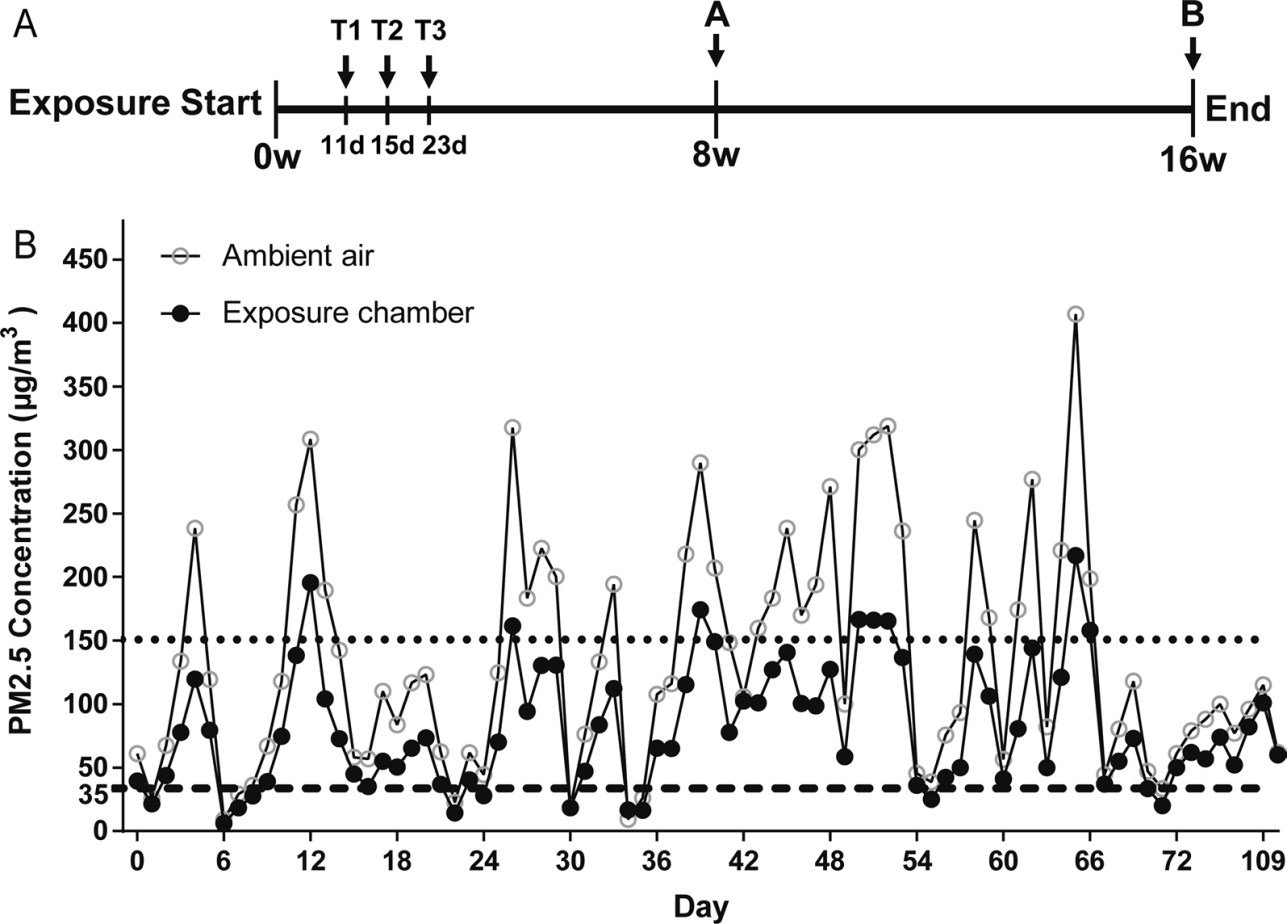
Experimental design of sub-chronic PM exposure. **A** The diagram of the experimental design of PM exposure in Shijiazhuang, China, 2019–2020. The arrows indicate the time points (days 11 (T1), 15 (T2), 23 (T3), 8 weeks (8w)) at which the C57BL/6J male mice were sacrificed. **B** The mean daily concentrations of PM2.5 in the exposure chamber (solid circle) and ambient air (hollow circle) over the duration of 16-week PM exposure. The dashed and dotted lines represent the mean daily limit and a level of severe air pollution according to the Air Quality Guideline of China

To mimic a real-ambient PM exposure scenario, we constructed a PM exposure system located in Shijiazhuang, China as described previously [5], and first conducted whole-body PM inhalation experiments in C57BL/6J male mice from November 27th, 2019 to March 26th, 2020 (Fig. 1A). The mean concentration of PM2.5 in ambient air and PM exposure chambers was 123.52 μg/m^3^ and 73.94 μg/m^3^, respectively, over a period of 16 weeks (Fig. 1B, Additional file 1: Table S1). To address the molecular mechanisms underlying the transition from acute pulmonary inflammation to chronic fibrotic changes, we sacrificed mice on days 11 (N = 10), 15 (N = 10), 23 (N = 10), 56 (8 weeks) (N = 20), and 112 (16 weeks) (N = 20), respectively, as representative time points of acute and sub-chronic PM exposures (Fig. 1A). In addition, 69 organic chemical components and 43 trace elements and metal species from collected PM2.5 were quantitatively analyzed (Additional file 1: Tables S2–S6). In summary, this exposure system mimics human exposure to the extent possible and the mice in the exposure chambers received a sustained high concentration of PM exposure.

**Fig. 2:**
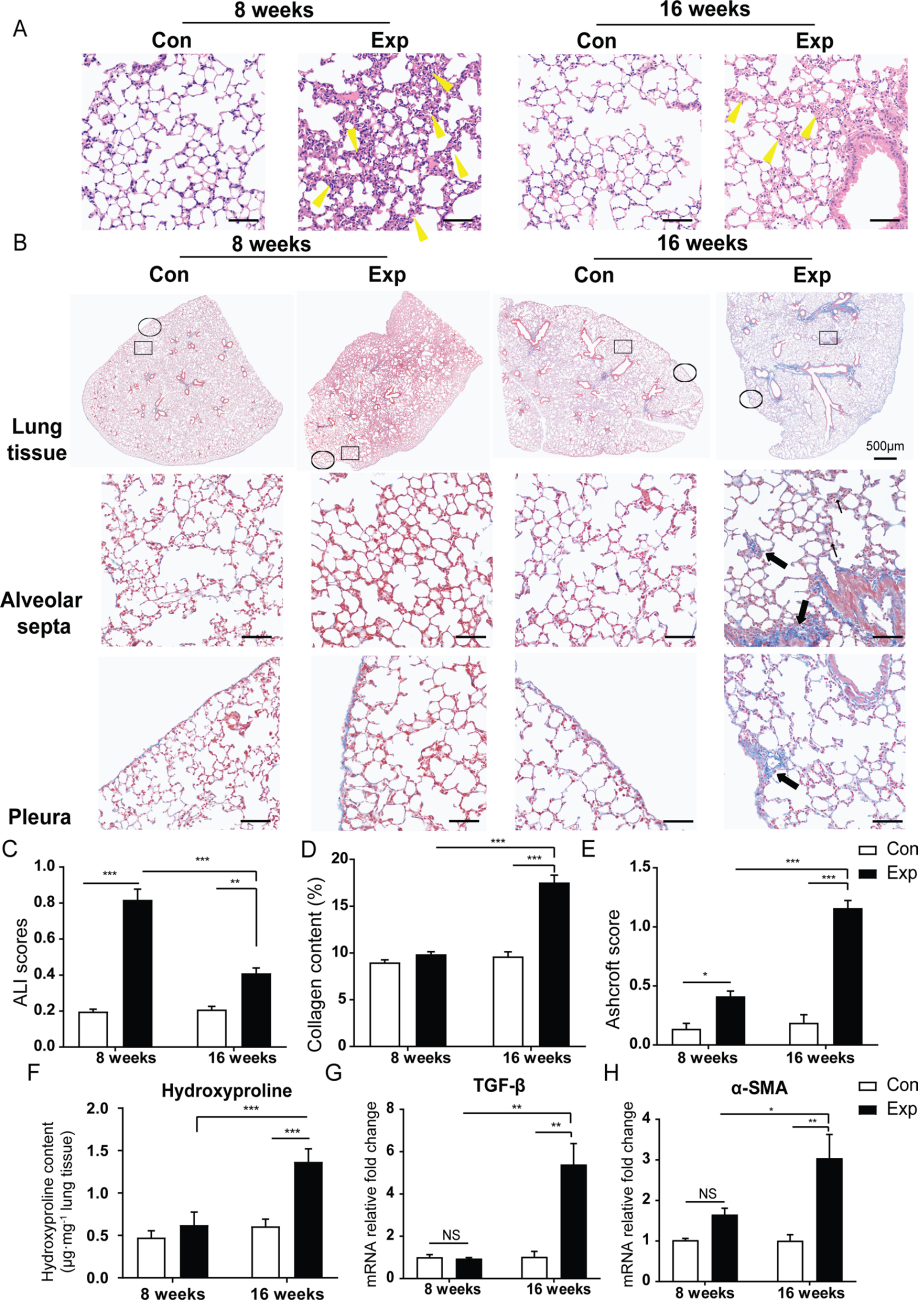
Effects of real-ambient PM exposure on chronic lung injury. Whole-slide images of lung sections stained with H&E in control and exposure group following 8-week and 16-week PM exposure. **A** Representative images of H&E-stained lung sections, displaying the pathological changes in the control and exposure groups following 8-week and 16-week exposure (scale bar = 50 μm). Yellow bold arrows indicate the interstitial neutrophils infiltration (N = 8). **B** Representative whole-slide images of lung sections stained with H&E in control and exposure group following 8-week and 16-week PM exposure (scale bar = 500 μm) and representative images of Masson's trichrome stained lung sections, showing the collagen deposition in different groups (scale bar = 50 μm). Black thin arrows indicate alveolar septa with gentle fibrotic changes and black bold arrows indicate the fibrotic changes with knot-like formation or pleural plaque formation. **C** ALI scores were calculated in different groups (N = 8). **D** Collagen content (%) in lung tissue was calculated as the ratio of labeled blue areas to total area of lung section (%/μm2 total area) upon Masson's trichrome staining (N = 8). **E** Ashcroft scores were calculated in the groups (N = 8). **F** Pulmonary hydroxyproline content in the control and exposure groups following 8-week and 16-week exposure (N = 3). **G**, **H** The relative mRNA expression levels of TGF-β, and profibrotic factor α-SMA in lung tissue of different groups (N = 5). Photographs were scanned by TissueFAXS analysis system. All magnifications are at 200X. Scale bar = 50 μm. The results were presented as mean ± SD. NS: Not significant; *P < 0.05; **P < 0.01; ***P < 0.001 compared with the control mice. Con: air-filtered control group; Exp: PM exposed group; ALI: acute lung injury

Consistent with previous observation [5], exposure to PM resulted in pathological changes. Acute inflammation was profound at 56 days (8 weeks) in mice following real-ambient PM exposure, while mild inflammation was present in mice placed in chambers installed with three layers of HEPA filters (Fig. 2A). As expected, chronic lung injuries were remarkable following 16-week PM exposure. Moreover, the chronic lung injuries were characterized by chronic inflammatory infiltrates, thickened alveolar septa with gentle fibrotic changes, isolated alveolar septa with gentle knot-like formations, moderate peri-bronchiolar fibrosis and pleural plaques due to excessive collagen deposition, as observed by Masson's trichrome and Sirius red staining (N = 8) (Fig. 2B, Additional file 1: Fig. S1). The acute lung injury (ALI) scores from H&E examination corroborated a more severe acute lung inflammation following 8-week versus 16-week exposure (Fig. 2C). The relative mRNA expression of pro-inflammatory cytokines, including IL-1β, IL-6, and IL-17A and anti-inflammatory cytokines such as IL-10 was 1.05- ~ 1.92-fold higher in mouse lung following 8-week PM exposure (N = 5) (all *P* < 0.05) (Additional file 1: Fig. S2A–F). The degree of fibrosis significantly increased in mouse lung tissue after 16-week PM exposure compared to the control group (*P* < 0.001) based on the increase in collagen content (%) and Ashcroft score [15] (Fig. 2D, E, Additional file 1: Table S7). In line with the pathological findings, we found that the content of hydroxyproline (Hyp) in lung tissue was 2.25-fold higher following 16-week exposure compared to that in control group (*P* < 0.001), while we failed to observe significant changes in lung tissue following 8-week PM exposure (N = 3) (Fig. 2F). In addition, the content of type 1 collagen in lung tissue only significantly increased in lung tissue of mice experiencing 16-week PM exposure (N = 3) (Additional file 1: Fig. S2G). Moreover, the expression of fibrosis-related genes, including Acta2, Tgfb1, Col1a1, Fibronectin and S100a4 were significantly increased in mouse lung tissues only after 16-week PM exposure (N = 5) (all *P* < 0.05) (Fig. 2G, H, Additional file 1: Fig. S2H–J). Collectively, the pathological fibrotic changes and relative biochemical indicators revealed the occurrence of initiation of mild pleural and interstitial pulmonary fibrosis accompanied with chronic inflammatory infiltrations following 16-week PM exposure. Here, we established a time-course animal model under real-ambient PM exposure, which provided us an ideal scenario to address mechanisms which trigger the transition from acute inflammation to chronic lung injuries and initiation of lung fibrosis.

### ScRNA-seq analysis reveals immune dysregulations and activation of IL-17A signaling pathway associated with PM-induced pulmonary fibrosis

**Fig. 3:**
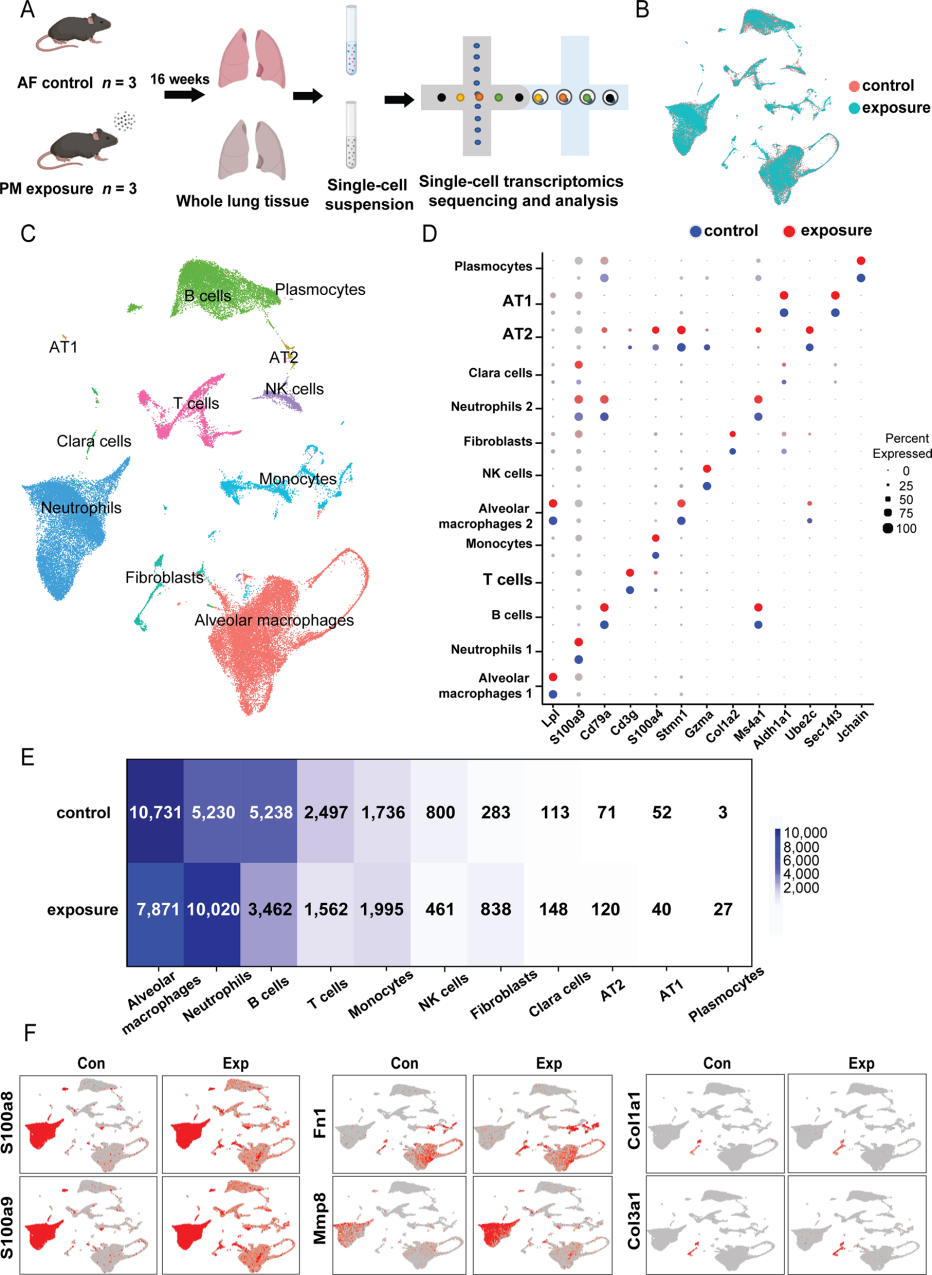
scRNA-seq of mouse whole lung cells in response to sub-chronic PM exposure. **A** Schematic diagram of single-cell samples preparation. **B** UMAP plot of whole lung cells in control and PM exposed groups. Cells from whole lungs of control or exposed group are indicated by red or turquoise color. **C** Thirteen clusters with 11 cell types identified on the UMAP plot. **D** The dot plot represents the expression level of the representative marker genes (dot color shades) and the percentage of cells expressing marker genes (dot size). **E** The heatmap illustrates the number of whole lung cells in control and PM exposure groups. **F** Expression levels of proinflammatory and profibrotic genes including S100a8, S100a9, Fn1, Mmp8, Col1a1, and Col3a1 on UMAP plots split by groups of different exposure status (N = 3). AT, alveolar epithelial cells; Con, control group; Exp, PM exposure group; UMAP, Uniform Manifold Approximation and Projection

To clarify the interactions between immune cells and stromal cells in response to sub-chronic PM exposure, we conducted scRNA-seq on whole lung samples isolated from control and sub-chronic PM exposure groups (N = 3) at the end of the 16-week exposure (Fig. 3A). With aggregation and the quality control of pre-processing, we obtained datasets of mouse lungs containing 26,745 and 26,532 cell profiles in control and PM exposure group, respectively (Additional file 1: Table S8). To characterize the whole lung cell atlas, we performed canonical correlation analysis (CCA) to integrate datasets with unsupervised clustering and visualization by Uniform Manifold Approximation and Projection (UMAP) (Fig. 3B). Using the online databases including PanglaodB and single-cell Mouse Cell Atlas (scMCA) available for annotations of specific cell markers, we were able to identify 11 major types of cells that fell into 13 cell clusters (Fig. 3C, D). Particularly, the immune cells, including alveolar macrophages (AMs, clusters 0 and 5), neutrophils (clusters 1 and 8), B cells (cluster 2), T cells (cluster 3), monocytes-derived cells (monocytes, cluster 4), NK cells (cluster 6) and plasmocytes (cluster 12) were the major cell populations in the lung tissue. As shown in Fig. 3D, we identified that pulmonary stromal cells were comprised mainly of fibroblasts (cluster 7), Clara cells (cluster 9), type 2 alveolar epithelial cells (AT2, cluster 10), and type 1 alveolar epithelial cells (AT1, cluster 11) (detailed information of markers were listed in Additional file 1: Table S9). Notably, following 16-week PM exposure, the number of major immune cells, including AMs, B cells, T cells, NK cells, and epithelial cells, decreased significantly, while the number of neutrophils, monocytes, and fibroblasts increased by 1.92-, 1.15-, and 2.96-fold, respectively, compared to the control group (Fig. 3E). Meanwhile, consistent with the scRNA-seq results, the number and proportion of total T cells significantly decreased (*P* < 0.001), but interstitial macrophages significantly increased (*P* < 0.001), which were confirmed by flow cytometry assay (Additional file 1: Fig. S3A). The differentially expressed genes (DEGs) analysis was conducted in each cell cluster (Additional file 2). We found all cell clusters in PM-exposed mouse lungs ubiquitously expressing higher levels of chronic inflammation markers, S100a8 and S100a9. The elevated expression of specific genes in relation to the synthesis of extracellular matrix (ECM) and tissue remodeling, including Fn1, Mmp9, Col1a1, and Col3a1 only appeared in three cell clusters including neutrophils, monocytes, and fibroblasts (Fig. 3F). Taken together, a significant increase in the number of fibroblasts and upregulation of profibrotic gene expression characterize the initial state of chronic fibrosis. Importantly, the decreased population of most immune cells implicates that immune dysregulation might be critical at the initial stage of pulmonary fibrosis upon sub-chronic PM exposure.

**Fig. 4:**
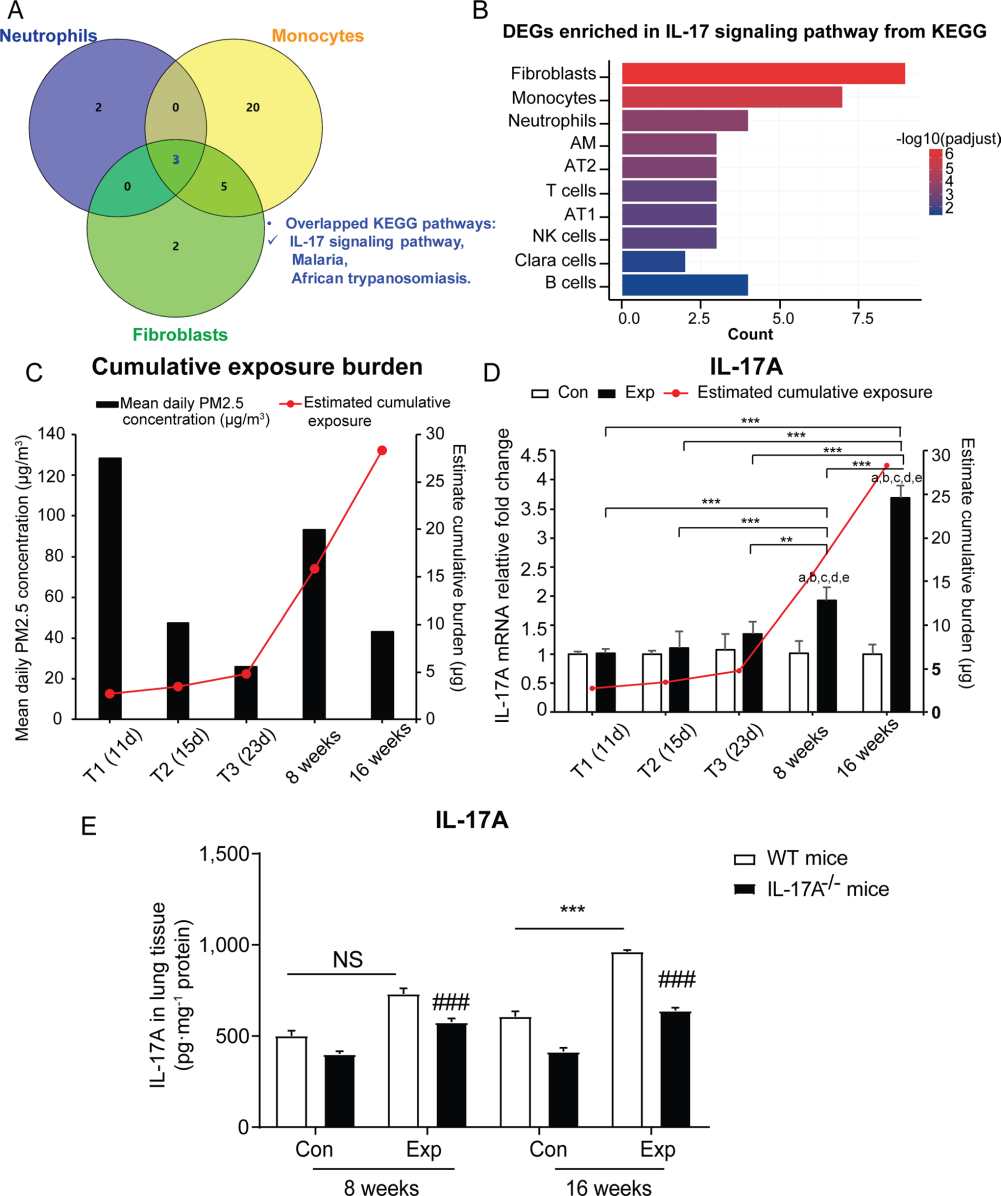
Activation of IL-17 signaling pathway is involved in PM-induced pulmonary fibrosis. **A** Venn diagram depicts the overlapping enriched KEGG pathways based on the DEGs identified in the cell clusters of neutrophils, monocytes and fibroblasts in control and PM exposure group by scRNA-seq. **B** IL-17 signaling pathway is emerged from analysis of enriched KEGG pathways among the respective DEGs in 10 cell clusters. **C** The mean daily PM2.5 concentration (μg/m^3^, as shown in left y-axis) and cumulative exposure burden in mouse lungs (μg, as shown in right y-axis) calculated by MMPD program. **D** IL-17A mRNA expression in lung tissue from control and PM exposed groups at T1 (11 d), T2 (15 d), T3 (23 d), 8-weeks (56 d) and 16-weeks (112 d), and the respective cumulative exposure burdens. Results were calculated as fold changes versus the corresponding control group (N = 5). The data are expressed as mean ± SD. **P < 0.01; ***P < 0.001 compared with PM-exposed mice at different time points. ^a^*P* < 0.001 compared with control mice at T1; ^b^*P* < 0.001 compared with control mice at T2; ^c^*P* < 0.001 compared with control mice at T3; ^d^*P* < 0.001 compared with control mice at 8 weeks; ^e^*P* < 0.001 compared with control mice at 16 weeks. **E** Contents of IL-17A in lung tissue from WT and IL-17A^−/−^ mice in control and PM exposed group following 8-week and 16-week exposure (N = 5). The data are expressed as mean ± SD. NS: Not significant; *P < 0.05; **P < 0.01; ***P < 0.001 compared with the control mice. ^###^*P* < 0.001 compared with the WT PM-exposed mice (IL-17A^−/−^-PM vs WT-PM). Con: control group; Exp: PM exposure group; DEGs: differential expressed genes

To identify the essential cytokines or chemotaxis factors that trigger the initiation of pulmonary fibrosis, we performed analysis on DEGs in each of the cell cluster using the GO Biological Process (GOBP) and Kyoto Encyclopedia of Genes and Genomes (KEGG) pathway enrichment programs (detailed information was listed in Additional file 3, Additional file 4). Notably, most of the DEGs were abundantly expressed in neutrophils, monocytes, and fibroblasts with an overlapping annotation of IL-17 signaling pathway (Fig. 4A, Additional file 1: Fig. S3B). The activation of IL-17 pathway was also identified using the Ingenuity Pathway Analysis (IPA) software (Additional file 1: Fig. S3C, D). Moreover, the IL-17 pathway was significantly enriched in various cell clusters (*P* < 0.05), indicating that the perturbation of IL-17 signaling was ubiquitous in pulmonary stroma (Fig. 4B). With the searching terms in GOBP program, the leukocyte chemotaxis and neutrophil migration in monocytes and fibroblasts, and the response to interferon-α/β/γ and collagen metabolic process in neutrophils, we showed that perturbation of IL-17 signaling pathway in these cell clusters might act as a mediator for recruiting immune cells in chronic inflammatory microenvironment upon sub-chronic PM exposure (Additional file 1: Fig. S4A–C). Taken together, we speculate that the activation of IL-17 signaling pathway might be involved in the development of PM-induced pulmonary fibrosis.

### IL-17 signaling pathway activation mediates PM-induced pulmonary fibrosis

To analyze cellular interactions based on scRNA-seq data, we focused on the top 20 significant changes in category ‘cytokines’ and discovered the enhanced interaction of Il17a and Il17rc within T cells, which might be responsible for the increased number of Th17 cells and secretion of IL-17A (Additional file 1: Fig. S5A, B and Additional file 5). As shown in Fig. 4C and Additional file 1: Table S1, the respective cumulative exposure burden of PM_2.5_ at 5 time-points increased, while the mean concentration varied. Next, we examined the level of IL-17A, which was formally regarded as IL-17, in mouse lung tissues isolated from different time points of T1, T2, T3, 8-week, and 16-week PM exposure (N = 5). No significant difference was observed in the secretion level of IL-17A at T1, T2, and T3 time points with less cumulative PM exposure burden. Even though the mean concentration of PM_2.5_ two days before the end of 16-week PM exposure was much lower than the time point of 8-week exposure group, the secretion of IL-17A gradually increased, starting at the end of 8-week throughout the end of 16-week exposure, showing a significant difference from the control group (Additional file 1: Fig. S5C). As the cumulative PM exposure burden increased over three time points (T3, 8-week and 16-week), the relative mRNA expression of IL-17A was significantly upregulated by 25.48%, 48.04%, and 72.84% in lung tissues (all *P* < 0.01), respectively (Fig. 4D). Importantly, we showed that the slightly increased IL-17A was produced by T cells and the specific receptor IL-17RA was distributed in most of the immune cells including AMs, neutrophils, monocytes, etc. (Additional file 1: Fig. S5D). In addition to elevated mRNA expression of IL-17A, the expression of IL-17 signaling downstream genes including p65, IL-1β, IL-6, S100a9, Cox2, and Ccr2 were only significantly upregulated at the time point of 16-week PM exposure (*P* < 0.05) (Additional file 1: Figs. S2B, D, S5E–H). Taken together, these findings reveal that the gradually rising of IL-17A occurs concomitantly with an increasing cumulative burden of PM exposure, implying that IL-17A plays an important role in the onset of pulmonary fibrosis.

**Fig. 5:**
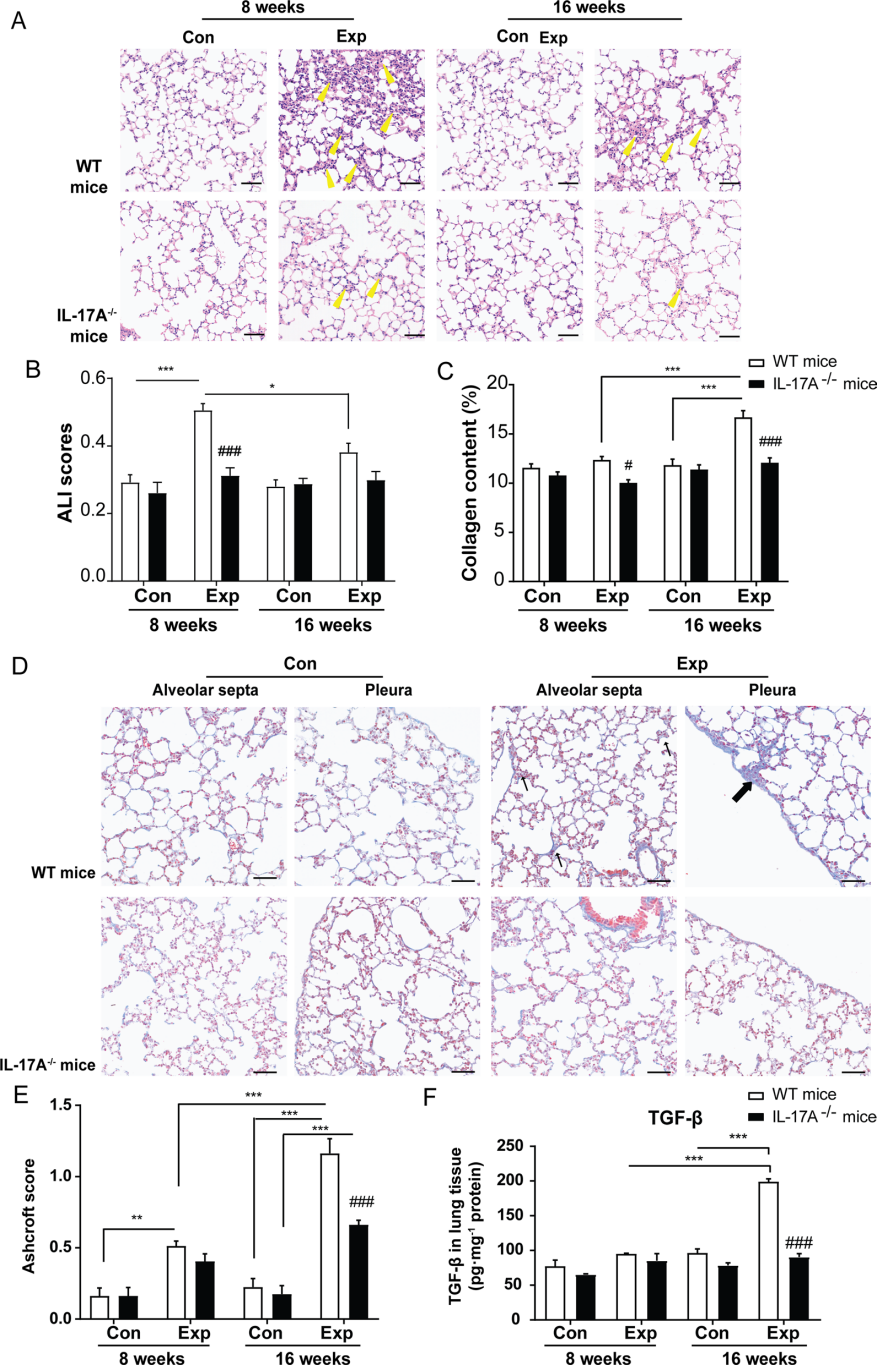
IL-17A deficiency attenuates PM-induced chronic lung injuries. **A** The inflammatory status of representative H&E-stained lung sections in 8-week and 16-week PM exposure groups and their corresponding control groups in WT and IL-17A^−/−^ mice. Yellow bold arrows indicate the interstitial neutrophils infiltration. **B** ALI scores assessed based on the pathological changes shown in **A** (N = 8). **C** Collagen content (%) in lung tissue was calculated as the ratio of labeled blue areas to total area of lung section (%/μm^2^ total area) upon Masson's trichrome staining (N = 8). **D** Representative Masson's trichrome-stained lung sections indicate the profibrotic changes including collagen deposition, knot-like formation, and thickened alveolar septum in 16-week PM exposure group from WT and IL-17A^−/−^ mice (N = 8). **E** Ashcroft scores were calculated in the WT and IL-17A^−/−^ mice in control and PM exposed groups following 8-week and 16-week exposure (N = 8). **F** The levels of TGF-β in lung tissue from WT and IL-17A^−/−^ mice in control and PM exposure groups following 8-week and 16-week exposure (N = 3). Photographs were obtained through scanning by TissueFAXS analysis system with the 200X magnification. Scale bar = 50 μm. The results are shown as mean ± SD. *P < 0.05; **P < 0.01; ***P < 0.001 PM-exposed mice compared with the control mice (WT^−^-PM vs WT-Con). ^#^*P* < 0.05 compared with the WT PM-exposed mice (IL-17A^−/−^-PM vs WT-PM); ^###^*P* < 0.001 IL-17A^−/−^-PM vs WT-PM. Con: control group; Exp: PM exposure group; ALI: acute lung injury

To further assess the fundamental role of IL-17A in mediating PM-induced pulmonary fibrosis, homozygous IL-17A^−/−^ mice and wildtype (WT) littermate (20 mice/group) were subjected to 8-week and 16-week PM exposure in real-ambient PM exposure system in the winter season of 2020. For quality control, we verified that the target exons were successfully knocked out and IL-17A expression was significantly decreased by 87% in IL-17A^−/−^ mouse lung (*P* < 0.001) (N = 5) (Additional file 1: Fig. S6A–C and Additional file 6). The level of IL-17A mRNA remained unchanged in splenic T cells derived from IL-17A^−/−^ mice after anti-CD3/anti-CD28 co-activation and the secretion level of IL-17A was undetectable in the supernatant (Additional file 1: Fig. S6D, E). As for WT mice, the mean content of IL-17A was 1.58 times higher than in the control group (*P* < 0.001) following 16-week PM exposure, while no significant difference was observed after 8-week exposure (N = 5) (Fig. 4E). Concomitant to the significant decrease in IL-17A secretion in IL-17A^−/−^ mice following PM exposure (N = 3) (Fig. 4E), we observed a 38.25% decline in acute inflammation based on the ALI score following 8-week PM exposure (N = 8), suggesting efficient attenuation of lung inflammation induced by PM exposure (Fig. 5A, B, Additional file 1: Table S10). The collagen content (%) and Ashcroft score in IL-17A^−/−^ mice following 16-week PM exposure were decreased by 27.22% and 43.01% (both *P* < 0.001), respectively (N = 8) (Fig. 5C–E, Additional file 1: Table S10). Meanwhile, we observed a 39.80% decrease in Hyp content in lung tissue of IL-17A^−/−^ mice following 16-week PM exposure compared to WT mice (Additional file 1: Fig. S6F). Collectively, depletion of IL-17A could efficiently attenuate chronic lung injuries induced by sub-chronic PM exposure.

It has been demonstrated that elevated TGF-β contributes to fibrogenesis in multiple organs [16]. To clarify the interplay between IL-17A and TGF-β in the development of progressive fibrosis, we collected mouse lung tissues from WT and IL-17A^−/−^ mice after 8-week or 16-week PM exposure. As described above, significantly increased secretion of IL-17A was noted in the lungs of WT mice following 16-week PM exposure compared to the control group (*P* < 0.001). The extent of increased IL-17A was correlated with the levels TGF-β in mouse lung after 16-week PM exposure, indicating that IL-17A and TGF-β might be cooperating in mediating pulmonary chronic inflammation. Notably, the secretion of TGF-β decreased by 20.9% or 52.0% in IL-17A^−/−^ mice after 8-week or 16-week PM exposure, respectively (Fig. 5F), indicating a significant role of IL-17 in regulating TGF-β secretion. Taken together, we reveal that activation of IL-17 pathway is indispensable in the course of the development of pulmonary fibrosis upon sub-chronic PM exposure through regulation of TGF-β signaling cascade.

### IL-17 signaling is involved in recruitment of immunosuppressive MDSCs

**Fig. 6:**
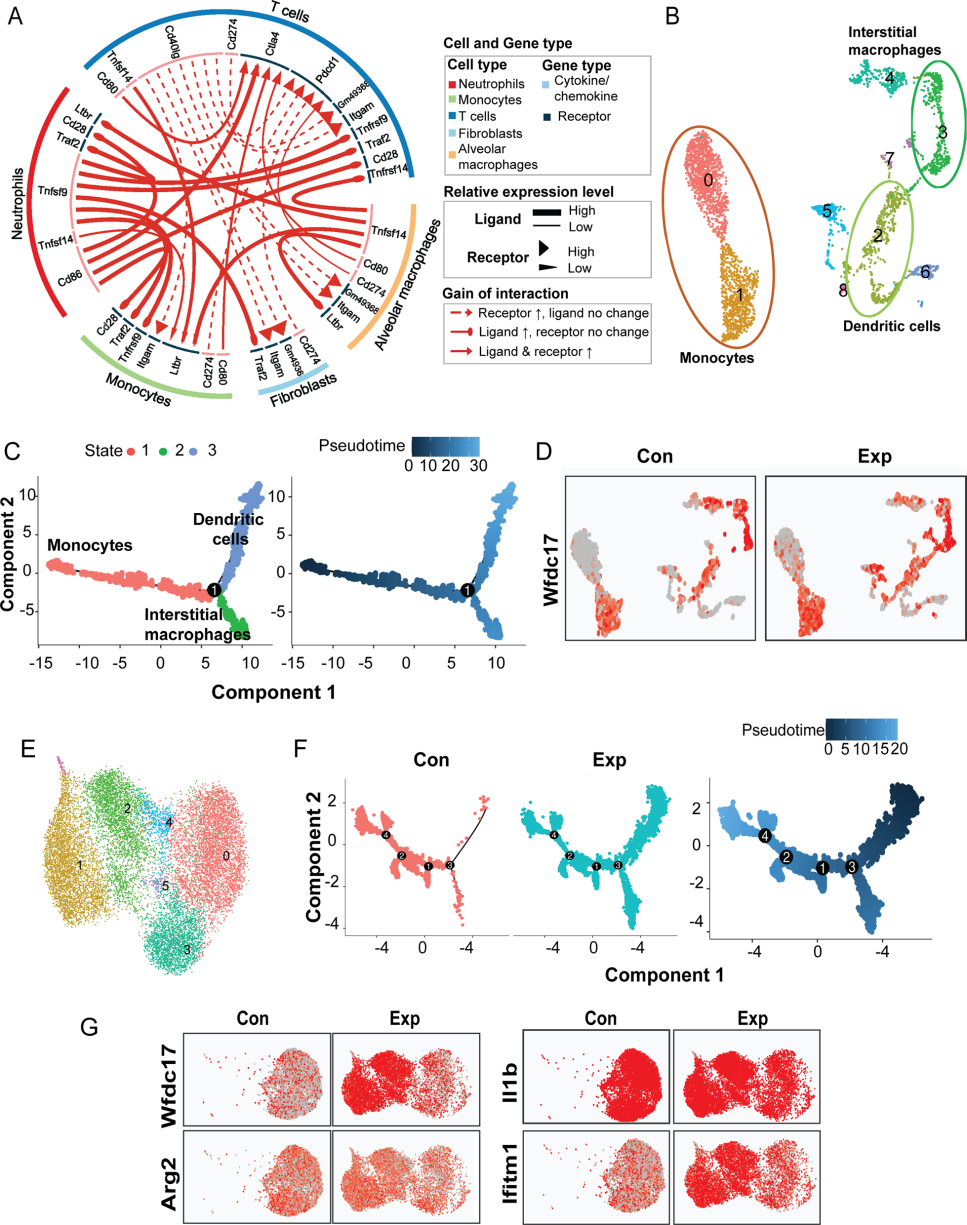
Identification of immunosuppressive cells in neutrophil and monocyte-derived cell subset following sub-chronic PM exposure. **A** Circos plot is depicted for significant alterations (PM exposure group vs control group) in cellular interactions among neutrophils, monocyte-derived cells (displayed as monocytes), fibroblasts, alveolar macrophages, and T cells. **B** Sub-clusters including monocytes (cluster 0 and 1), dendritic cells (cluster 2), and interstitial macrophages (cluster 3) are identified from the UMAP plot of monocyte-derived cell subset. **C** Pseudotime trajectory analysis in the left panel indicates the differentiation state of the monocyte-derived cells as shown in red (monocytes), green (interstitial macrophages), or blue (dendritic cells). The other one in the right panel was colored from dark blue to light blue, indicating pseudotime scores from the lowest to the highest in monocyte-derived cells. **D** Each dot represents the expression of Wfdc17 in the UMAP plots from the monocyte-derived cell subset, colored from grey to red, indicating the low to high expression. Cells from control and PM exposed group were shown in the separate column. **E** Sub-clusters are identified in neutrophil subset on UMAP plot. **F** The results of pseudotime trajectory analysis were split by exposure status (left panel) and colored based on pseudotime scores (right panel) in neutrophil subset, from dark blue to light blue, indicating from the lowest to the highest score. **G** The expression of Wfdc17, Arg2, Il1b, and Ifitm1 was shown in UMAP plots from control and PM exposed groups, which was displayed by the colors from grey to red. Cells from control and PM exposed groups were shown in the separate columns. Con: control group; Exp: PM exposure group; UMAP: Uniform Manifold Approximation and Projection

To further address the impact of the perturbation of IL-17 signaling on immune dysregulation, firstly we elaborated the immune dysfunctions by analyzing the scRNA-seq dataset. Of the top 20 significant pairs of ligand-receptor that regulate the cell–cell interaction, the cytotoxic T lymphocyte activation-4 (Ctla4) was activated by enhanced ligand-receptor pairs Cd86-Ctla4 between neutrophils and T cells, or Cd80-Ctla4 between monocytes and T cells, indicating that the differentiation of immunosuppressive regulatory T cells (Tregs) might be mediating chronic inflammation upon PM exposure (Fig. 6A, Additional file 5).

Next, we analyzed the specific markers for extracted subsets in neutrophils or monocytes cluster and revealed that myeloid-derived suppressor cells (MDSCs) were involved in regulation of immune response by suppressing T cells upon PM exposure. Two subtypes of MDSCs, polymorphonuclear myeloid-derived suppressor cells (PMN-MDSCs) and monocytic myeloid-derived suppressor cells (M-MDSCs) share the same myeloid progenitors of neutrophils and monocytes. With specific markers, we annotated cluster 0 and cluster 1 as monocyte progenitors or immature monocytes, cluster 2 as dendritic cells, and cluster 3 as interstitial macrophages (Fig. 6B, detailed information was provided in Additional file 1: Table S11). The pseudotime trajectory analysis and pseudotime score calculated by Monocle package proposed a chronological order of differentiation from cluster 0 into cluster 1 (State 1), followed by dendritic cells (State 2) and interstitial macrophages (State 3) as two branches of terminal states (Fig. 6C, D). With respect to the neutrophil subset, cluster 1 in the state of the lowest pseudotime scores increased in response to PM exposure (Fig. 6E, F and the detailed information of top 10 markers and cellular proportions in different groups were provided in Additional file 1: Table S12). Moreover, gene expression of Wfdc17, Arg2, Il-1β, and Ifitm1, which have been implicated in immunosuppressive effects, was greatly upregulated in this sub-cluster of neutrophils (Fig. 6G). The pseudotime analysis revealed that sub-clusters in the early and transitional states within neutrophilic and monocytic lineages exhibited enhanced immunosuppressive activity, which might be carried out by MDSCs.

**Fig. 7:**
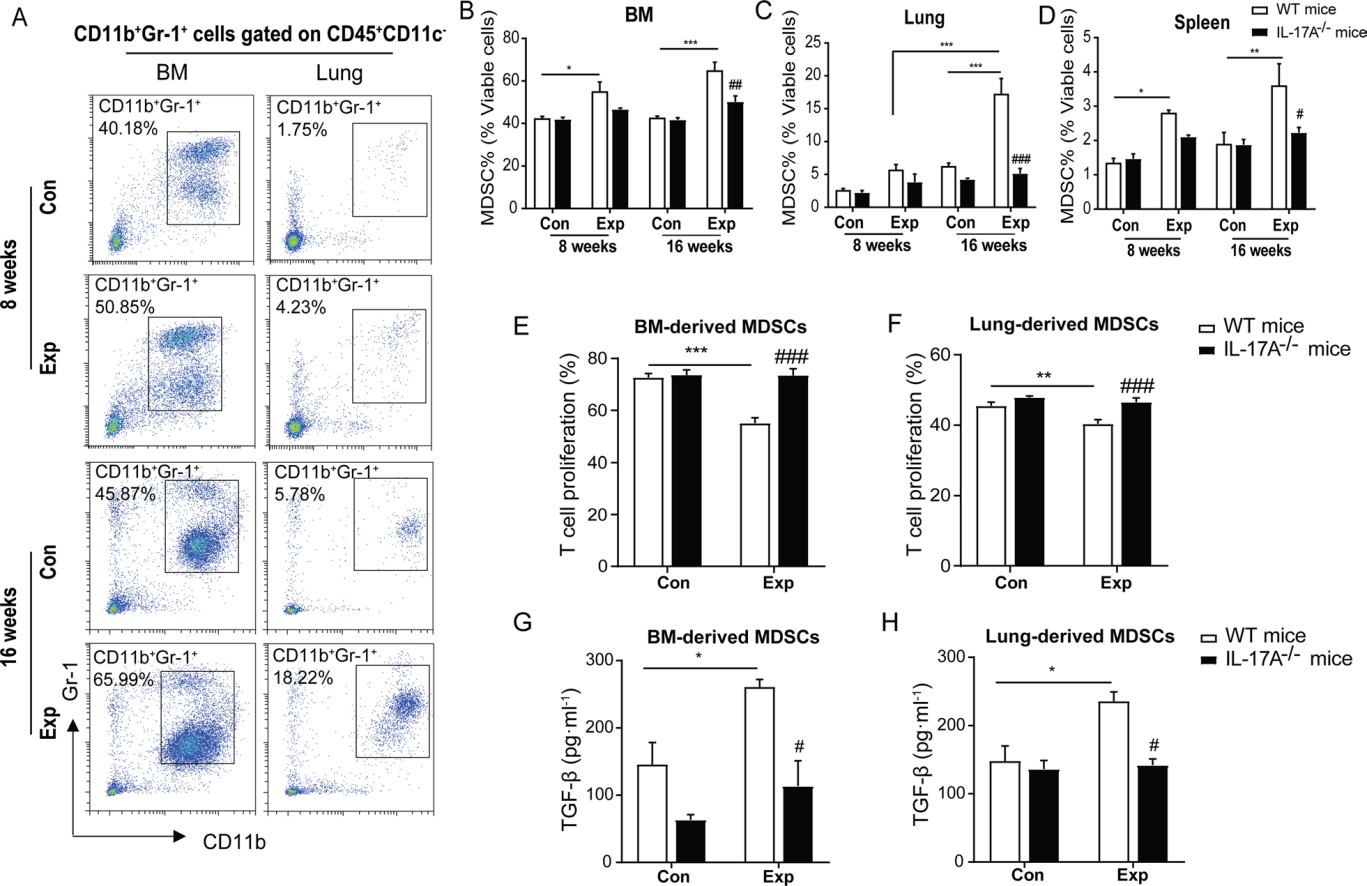
Immunosuppressive MDSCs recruited by elevated IL-17A after sub-chronic exposure to PM. **A** Gating strategy of the flow cytometry for MDSCs detection (CD11b^+^Gr-1^+^ cells) in the bone marrow and lung tissues from 8-week and 16-week PM exposed groups and their corresponding control groups. The gated CD11b^+^Gr-1^+^ cells show an increasing MDSCs proportion in both bone marrows and lung tissues following 8-week and 16-week PM exposure. **B**–**D** The proportions of MDSCs detected in the viable cells of bone marrows (**B**), lung tissues (**C**) and spleens (**D**) in control and PM exposed groups from WT and IL-17A^−/−^ mice (N = 4). **E**, **F** Immunosuppressive effects of MDSCs derived from 16-week PM exposed group on T cell proliferation (%) appeared after coculturing MDSCs derived from bone marrow (**E**) and lung (**F**) with T cells at the ratio of 1:2 in vitro. (G-H) TGF-β levels in the supernatant of the coculture system after splenic T cells were cocultured with MDSCs from bone marrow (**G**) and lung (**H**) for 72 h. The experiment was carried out in 3 replicates. The results are presented as mean ± SD. *P < 0.05; **P < 0.01; ***P < 0.001 PM-exposed mice compared with the control mice (WT^−^-PM vs WT-Con). ^#^*P* < 0.05 compared with the WT PM-exposed mice (IL-17A^−/−^-PM vs WT-PM). ^###^*P* < 0.05 compared with the WT PM-exposed mice (IL-17A^−/−^-PM vs WT-PM). Con: control group; Exp: PM exposure group

In addition, we performed flow cytometry assay to distinguish the subtypes of MDSCs from mouse lungs and bone marrows isolated at the end of 8-week and 16-week PM exposure (N = 4). As shown in Fig. 7A and Additional file 1: Fig. S7A, the markers CD11b^+^Gr1(Ly6G/Ly6C)^+^, CD11b^+^Ly6C^−^Ly6G^hi^, and CD11b^+^Ly6C^+^Ly6G^int^ were used for labeling total MDSCs, PMN-MDSCs, and M-MDSCs, respectively. We found a significant increase in the proportion of MDSCs in viable cells by 81.37%, respectively, from mouse bone marrow especially following 16-week PM exposure (*P* < 0.001) (Fig. 7B). Moreover, there was an increasing trend in number of MDSCs recruited into mouse lung interstitium with the increase of exposure time (*P* < 0.001) (Fig. 7C). The proportion of both PMN-MDSCs and M-MDSCs subsets were significantly increased in bone marrows and lung tissues of mice undergoing 16-week PM exposure (*P* < 0.05). However, the significant difference appeared in M-MDSCs subset only in bone marrow following 8-week PM exposure (*P* < 0.05) (Additional file 1: Fig. S7B–G). To further address whether IL-17A was critical for MDSCs recruitment, we examined the amounts of MDSCs in IL-17A^−/−^ mice following real-ambient PM exposure. As shown in Fig. 7B–D, the number of MDSCs significantly declined by 22.76% in mouse bone marrow, by 38.23% in mouse spleens, and by 70.27% in mouse lungs (all *P* < 0.05) of IL-17A^−/−^ mice following 8-week and 16-week PM exposure compared to those in WT mice, indicating that the recruitment of MDSCs in lung might be regulated by IL-17A signaling. These results suggest that IL-17 signaling activation is prerequisite for recruitment of MDSCs in lung upon PM exposure, leading to alterations in the microenvironment and favoring the development of chronic inflammation.

Furthermore, the immunosuppressive effect of recruited MDSCs was examined by ex vivo proliferation functional assay. The results showed a 17.62% and 5.12% suppression by MDSCs derived from WT mouse bone marrow and lungs after exposure to PM for 16 weeks compared to the controls (all *P* < 0.05), indicating that chronic inflammation induced by PM exposure conferred an immunosuppressive state carried out by MDSCs. Notably, the immunosuppressive activities of both bone marrow and lung MDSCs were significantly attenuated (18.48% and 6.15% in bone marrow and lungs, respectively) in IL-17A^−/−^ mice following 16-week PM exposure compared to WT littermates (all *P* < 0.001) (Fig. 7E, F). Corroborating the in vitro observations of enhanced immunosuppressive activity of MDSCs, we found that the proliferations of total T cells in lung tissue were significantly suppressed after 8-week or 16-week PM exposure (both *P* < 0.01) (Additional file 1: Fig. S3A). In parallel, by conducting in vivo MDSCs depletion experiment, we noticed that the decline of MDSCs attenuated the transition from acute to chronic lung injury, demonstrating the potential profibrotic role of MDSCs in the progression of pulmonary fibrosis (Additional file 1: Fig. S8A–E). Collectively, we conclude that the increased recruitment of MDSCs by activation of IL-17A in response to PM exposure exhibited enhanced immunosuppressive potential that might be involved in initiation of fibrosis through elevating TGF-β production.

### Activation of IL-17A pathway triggers the initiation of pulmonary fibrosis through altering microenvironment comprising of excessive TGF-β secretion and defective macrophages functions

Notably, we showed that enhanced immunosuppressive activity of MDSCs was related to higher level of profibrotic factor TGF-β in a coculture system engaging MDSCs and T cells, where MDSCs were isolated from mice receiving 16-week PM exposure compared to the coculture model with MDSCs isolated from the control mice or T cells (*P* < 0.05). Additionally, concomitant with the weaker immunosuppressive activity of MDSCs derived from bone marrow and lung of IL-17A^−/−^ mice, there was a 56.45% and 39.70% decrease in the secreted TGF-β in supernatant of the coculture system (*P* < 0.001) (Fig. 7G, H). These findings enhanced the notion that increased IL-17A participates in generating and recruiting immunosuppressive MDSCs which might be responsible for regulating of TGF-β secretion in the lung microenvironment. Key events in the initiation of pulmonary fibrosis include fibroblast activation, increasing synthesis of collagen, and impeded ECM degradation. We analyzed a unique cluster of fibroblast subset, containing fibrocytes derived from bone marrows that expressed abundantly higher levels of Ccl2 and Fn1 in mouse lungs in PM exposed group (Additional file 1: Fig. S9A–C). Upregulation of Ccl2 in lung induced by one of the immunosuppressive cytokines IL-10 has been previously demonstrated to mediate the recruitment of fibrocytes [17] and promote the differentiation of activated fibroblasts and myofibroblasts especially under the microenvironment with excessive TGF-β [18]. Notably, these activated fibroblasts were able to proliferate and synthesize collagen. To further clarify the role of increasing interstitial macrophages involved in mediating immunosuppression, we conducted informatics analysis on the scRNA-seq data and perform ex vivo functional studies. We found that the increased population of interstitial macrophages partially originated from M-MDSCs in response to PM exposure. The upregulated DEGs with M2 profibrotic annotation, including S100a9/S100a8, S100a4, Chil3 (encoding YM-1), and Arg1 were increased in mouse interstitial macrophages following 16-week PM exposure, suggesting the potential defective macrophage functions (Additional file 1: Fig. S9D–G). Collectively, these findings indicate that MDSCs recruited by activated IL-17A might work in synchrony with the activation of fibroblasts to perturb the balance between collagen synthesis and clearance, leading to the initiation of pulmonary fibrosis in response to PM exposure.

## Discussion

The latest released 2021 WHO annual report declared that sustained sub-chronic ambient air PM_2.5_ exposure poses threat to human health [19, 20]. Short- and long-term exposures have been linked to chronic respiratory and heart diseases [21]. Although great efforts have been made to elaborate the mechanism of acute lung injury, less is known about the disrupted immune network involved in PM-induced chronic lung injuries. In this study, we addressed the molecular interactions among various pulmonary cells, which contributes to the transition of acute-to-chronic inflammation in response to real-ambient PM exposure. Our novel findings showed that sub-chronic PM exposure led to progressive pulmonary fibrosis, accompanied by gradual increasing in collagen deposition and significant changes in various cell types, as revealed by scRNA-seq analysis. Notably, we identified that activation of the IL-17 signaling pathway mediated, at least in part, PM-induced pulmonary fibrosis by regulating pulmonary TGF-β secretion and recruitment of immunosuppressive MDSCs. The interplay between distinct immune cells and altered microenvironment facilitated the proliferation and activation of fibroblasts. These findings provide new insight into the mechanisms by which the dysregulation of immune network attributable to PM-induced pulmonary fibrosis.

Chronic respiratory diseases, including pulmonary fibrosis, COPD, and asthma are associated with PM exposure in epidemiological studies [20, 22], and their mechanisms have been investigated in animals exposed to the concentrated ambient particles (CAP) by inhalation or by intratracheal instillation. With the advent of a whole body real-ambient and around-the-clock PM inhalation system [5, 6], we were able to mimic the natural state of human exposure to the extent possible. Moreover, the exposure mode of 24 h/day, 7 days/week leaves no intermittent time gap for recovery, as we have previously demonstrated [5]. In addition, absent further processing, the physiochemical properties of PM were maintained in their natural state. Specifically, the metal pipe heated by auto-temperature control system might cause deposition of the particles and the decrease in PM concentration in the chamber. However, we have confirmed that the particles with larger diameters were more likely to deposit in the pipe and thus fine particles were predominant in the exposure chamber [5]. The winter season in Shijiazhuang, in which we have chosen to conduct real-ambient PM exposure in winter for two years, ensures the sustained high PM levels in the chamber throughout the exposure duration. Collectively, this ideal exposure system is optimal for addressing the transition from continuous acute inflammation to chronic fibrosis over a course of sub-chronic PM exposure with minimum distress.

Due to the heterogeneity of lung tissue, gene expression patterns in sensitive cell types and the interactions between immune cells and stromal cells such as AMs [23], alveolar epithelial cells [24] and fibroblasts [25] might be masked and averaged with the signals from the infiltrating inflammatory cells in bulk RNA-seq analysis. To date, scRNA-seq analysis has been carried out in investigations of lung development [26], xenobiotics-induced pulmonary fibrosis [27, 28], and pulmonary aging [29], leading to comprehensive characterization of dynamic cellular composition, identification of specific rare cell types, and clarification of cell–cell interactions in the whole lung tissues at different states of diseases. Here, we utilized scRNA-seq to elucidate the potential immune mechanisms of initiation of pulmonary fibrosis following PM exposure. We show that the state of immunosuppressive microenvironment is prerequisite for the initiation of pulmonary fibrosis. Notably, we demonstrate that activation of the IL-17 signaling pathway in several cell cluster mediated activation of fibroblasts and transition from acute to chronic inflammation.

IL-17A, as a predominant upstream regulator of IL-17 signaling pathway promotes the progression of chronic inflammation-related pulmonary diseases, such as COPD and pulmonary fibrosis [30]. It orchestras with the other pro-inflammatory cytokines such as IL-6 and TNF-α to amplify the inflammation signaling induced by different xenobiotics [30, 31]. However, less is known how IL-17A contributes to the chronic lung injuries upon persistent high levels of PM exposure. In this study, we showed that the elevated IL-17A, accompanied with increased IL-1β, IL-6 and IL-10 in lung were associated with PM-induced pulmonary inflammation and collagen deposition, implicating a significant regulatory role of IL-17A on immune network by modulating both pro-inflammatory and anti-inflammatory cytokines. Moreover, it has been reported that increased IL-17A level mediates the resolution of inflammation after a single acute exposure, while persistent activation of IL-17A/IL-17RA axis upon repeated cellular injuries fails to resolve inflammation and impels tissue remodeling process [32]. However, whether and how IL-17A mediates the transition from acute to chronic lung injury remain unknown in the context of the progression of pulmonary fibrosis induced by sub-chronic PM exposure. Here, we demonstrate that statistically significantly increased IL-17A levels were only observed when acute inflammation transited to chronic inflammation with excessive collagen deposition gradually aggregating, while no significant increase was detected following short-term acute high-PM exposure. These results advance the possibility that IL-17A could be one of the potential biomarkers that indicates the key signaling mediating chronic inflammation and pulmonary fibrosis after experiencing sub-chronic persistent PM exposure.

In the pathological status of chronic inflammation, the immunosuppressive microenvironment, characterized by increased regulatory immune cells and anti-inflammatory cytokines could be involved in the promotion of pulmonary fibrosis induced by exposure to xenobiotics, including bleomycin [33] and micro- or nano-particles [34, 35]. With the utilization of scRNA-seq analysis, we were able to characterize the immunosuppressive microenvironment by identifying MDSCs subsets from highly increased number of neutrophilic and monocytic lineages in the fibrotic lungs induced by PM exposure. It has been demonstrated that IL-17A functions in cooperation with other cytokines including IL-6, GM-CSF, and IL-8 to regulate the immune microenvironment by enhancing myeloid-derived cells recruitment [33, 36], as well as facilitating tissue remodeling [32, 37]. The assumption that IL-17A participates in recruiting immunosuppressive MDSCs is supported by previous studies investigating the regulatory mechanism of autoimmune disease encephalomyelitis [38]. Blockade of IL-17A could effectively reverse the immunosuppressive state [33, 39]. Here, we show that the interplay between IL-17A and MDSCs plays an important role in promoting PM-induced pulmonary fibrosis. Notably, we confirmed that IL-17A enhanced the recruitment of immunosuppressive MDSCs and consequently, led to reduction of activated immune cells in the chronically inflammatory lung tissue. These results corroborate previous studies on bleomycin-induced pulmonary fibrosis [40] and parasite-driven cardiac fibrosis [41]. Further studies need to be conducted to better characterize the regulatory role of IL-17A on the interaction between MDSCs and activation of fibroblasts under the circumstances of immunosuppression in response to chronic PM exposure.

The profibrotic factor TGF-β has been recognized as one of the key events in the development of pulmonary fibrosis [42]. With its potential immunosuppressive activity [43], elevated TGF-β and MDSCs population function in cooperation to generate an immunosuppressive microenvironment. However, the interaction between IL-17A and TGF-β in development of pulmonary fibrosis remains unclear. It has been revealed that in the progression of liver fibrosis, IL-17A functions together with IL-22 to enhance TGF-β in hepatic stellate cells [44]. Other findings report that TGF-β regulates the differentiation of Th17 cells, which specifically affect the secretion of IL-17A at the early stage of inflammation and pulmonary fibrosis [45]. Importantly, IL-17A may exert the profibrotic role through TGF-β-dependent or -independent mechanisms in the progression of xenobiotics-induced pulmonary fibrosis [46]. Here, we demonstrate that IL-17A is involved in regulating and amplifying TGF-β signaling particularly in mediating chronic inflammation. We also reveal that MDSCs recruited by elevated IL-17A suppress the proliferation of T cells and simultaneously excessive TGF-β following sub-chronic PM exposure. It is likely that a microenvironment enriched with high content of TGF-β will lead to the alternatively activated macrophages M2 and proliferated fibroblasts [47]. In line with these findings, we identified that a portion of M2 macrophages were originated from MDSCs with less phagocytic activity, indicating a role of the cooperation between M2 macrophages and MDSCs in the maintenance of IL-17A-triggered immunosuppressive microenvironment. Taken together, we conclude that the activation of IL-17A regulatory pathway perturbs the immune balance by recruitment of MDSCs, in combination of the increasing TGF-β secretion, which contribute to the PM exposure-induced chronic inflammation.

## Conclusion

In conclusion, we illustrate that sub-chronic exposure to high concentration of PM leads to the initiation of pulmonary fibrosis. We describe the interplay between different cell clusters in response to PM exposure. Activated IL-17 signaling pathway appeared in many of cell clusters, providing the profibrotic and immunosuppressive microenvironment which is composed of elevated MDSCs, increased TGF-β, impaired macrophage phagocytosis, and activated fibroblasts in lung tissue. Thus, as the key modulator of pulmonary immune network, IL-17 signaling pathway interacts with other stromal cells and contributes to pulmonary fibrosis upon sub-chronic PM exposure. These findings provide novel insights into the mechanism of the environmental factors’ exposure induced human diseases.

## Methods

### Animals and real-ambient PM exposure

All animal procedures were approved by the guidelines of the Animal Care and Protection Committee of Sun Yat-sen University and Hebei Medical University. To better observe the chronic lung injuries and early pulmonary fibrosis, six-week-old male C57BL/6J mice were chosen and purchased from Beijing Vital River Laboratories. We conducted the real-ambient PM exposure experiments during winter season in November to March next year from 2019 to 2021. For real-ambient PM exposure, mice in each group (N = 20) at each time point of 8-week and 16-week PM exposure, 5 mice/cage) were housed in isolated ventilated cages (IVC) linked to the real-ambient PM exposure system that we installed in Shijiazhuang City, Hebei Province, China [5]. In this system, circulating fresh ambient air without concentrated PM is instilled into the chambers. The air channels are equipped with or without a three-layer HEPA filter in the control and exposure groups, respectively. During the 16 weeks of PM exposure, PM2.5 concentration, temperature, airflow rate, pressure, ventilation rate, humidity, noise and potential harmful microorganisms were constantly monitored. We monitored the PM2.5 concentration in ambient air and exposure chambers using Aerosol Detector DUSTTRAKTM II (TSI Incorporated, Shoreview). Notably, PM2.5 and PM1 were undetectable in the control chambers. The cumulative exposure burden of mouse lungs was calculated using Multiple-Path Particle Dosimetry (MPPD) program (version 3.04, Applied Research Associates, Inc). Ambient PM2.5 was collected onto Teflon filters and subjected to components analysis (detailed protocols was described in [5]). Simultaneously, a separate experiment for investigating PM exposure at 3 different time points (11, 15, 23 days) was conducted. Each group of mice were sacrificed on days 11, 15, 23, 56 (8 weeks), and 112 (16 weeks), respectively, following PM exposure. Three mice from the PM exposure and control groups at the end of 16-week exposure were randomly selected for scRNA-seq. Six-week-old C57BL/6 IL-17A^−/−^ male mice were kindly provided by Professor Yuan Zhuang from The Third Military Medical University [48], which were originally generated by Dr. Yoichiro Iwakura [49]. IL-17A^−/−^ mice and their WT littermates were exposed to HEPA-filtered air or real-ambient PM exposure for 8 weeks and 16 weeks (N = 20 at each time point). At each time point, mice were anaesthetized with 100 mg/kg sodium pentobarbital, the plasma and bronchoalveolar lavage fluid (BALF) was separated. The mouse lungs were fixed, snap-freeze in liquid nitrogen, or prepared as suspensions for further experiments.

### Histopathological analysis of lung tissues

The mouse lungs (N = 8) were perfused by ice-cold PBS and fixed in 4% paraformaldehyde (PFA) overnight at room temperature. Five μm-thick sections of paraffin-embedded lung tissues were placed on slides and subjected to hematoxylin–eosin (H&E), Masson's trichrome, and Sirius red staining. The entire sections were scanned using TissueFAXS (Tissuegnostics) and the ratio of labeled blue area to total area of lung section (%/μm^2^ total area) was automatically calculated upon Masson's trichrome staining using HistoQuest (Tissuegnostics) to indicate collagen content (%) in lung tissue. The extent of ALI was evaluated and scored according to the published American Thoracic Society report [50]. Both the collagen content (%) indicated by blue area (%/μm^2^ total area) and modified Ashcroft score system were utilized to evaluate the extent of pulmonary fibrosis.

### Hydroxyproline and collagen determination

Hydroxyproline and type I collagen contents in lung tissues were examined by Hydroxyproline Assay Kit and Collagen type I Assay Kit (Nanjing Jiancheng Bioengineering Institute).

### Lung dissociation and preparation of single cell suspension

Lungs from both groups (N = 3) were immediately perfused with PBS and harvested in the PBS supplemented with 2% fetal bovine serum (FBS, Gibco) and 1% penicillin–streptomycin (P/S, Thermo Fisher). The lungs were disassociated in cold digestion medium (Dulbecco's Modified Eagle Medium (DMEM, Gibco) with 1 mg/mL Collagenase/Dispase (Roche) and 0.1 mg/ml DNase I (Sigma Aldrich)) and mechanically minced into 1mm^3^ pieces. Then the suspension was incubated at 37 °C for 40 min with continuous shaking and later filtered through 70 μm cell strainers (Falcon BD). After centrifugation at 350× *g*, 10 min at 4 degrees, the cell pellets were suspended in red blood cell lysis buffer for 5 min and passed through 40 μm cell strainers (Falcon BD) to remove doublets or dead cells. Finally, the single-cell suspensions were resuspended in PBS supplemented with 0.5% bovine serum aluminum (BSA) at a density of 1 × 10^6^ cells/ml for further experiments.

### ScRNA-seq library preparation and sequencing

At the end of 16-week PM exposure, 6 mice in total (N = 3) were used for scRNA-seq. Approximately 10,000 cells were captured in each qualified sample with > 80% viable cells and the libraries were prepared according to the manufacturer’s protocol with Chromium Single Cell 3′ v3 Reagent Kit (10× Genomics). The libraries were sequenced on NovaSeq6000 (Illumina) at the sequencing depth of 500 M reads per cell. 6 samples were prepared, loaded and sequenced separately without a pooling. Detailed information about estimated cell counts and the reads mapped confidently to genome in each sample are summarized in Additional file 1: Table S7.

### ScRNA-seq data processing

Cellranger count function in Cellranger pipelines (version 3.0, 10× Genomics) was applied for analysis of alignment, filtering, barcode counting, and UMI counting. 3 samples from the same group were combined and analyzed the data using Cellranger aggr function. The expression matrixes were analyzed by Seurat R package (version 3.1). For quality control, cells with < 500 detected genes or > 6500 detected genes, and > 10% mitochondria genes were excluded from further analysis. Genes detected only in less than 3 cells were also eliminated. Data normalization and standardization were carried out and 2000 highly variable genes (HVGs) were identified using FindVariableGenes function for downstream integration and analysis. To examine the impact of PM exposure on specific cells, we integrated the scRNA-seq data using CCA provided by Seurat package. Dimensionality reduction was accomplished by principal components analysis (PCA) and clustering and visualization were carried out by utilizing UMAP using RunUMAP function of Seurat (principal components nPCs = 50 and resolution value 0.05). The top 10 conserved cell type markers across different conditions were identified by utilizing FindConservedMarkers functions. Cell types for each cluster were manually annotated based on the published literature, online database and automatic annotations from SingleR R package (version 4.0). Feature plots, dot plots and violin plots for the most representative markers in each cell cluster were carried out to confirm the specificity and discriminative power of the conserved markers by FeaturePlot and DotPlot functions. After confirming the cell types, we compared the actual number and proportion of cells in the different groups.

### DEGs identification and pathway enrichment analysis

Statistically significant and differentially expressed genes (log2|FC|> 0.25, *P* < 0.05, as the standard workflow) of different cell types in response to sub-chronic PM exposure were identified for each cell type using FindMarkers function. Pathway enrichment analysis was performed based on the generated DEGs of cell types utilizing Gene Ontology (GO), KEGG and Ingenuity Pathway Analysis (IPA) software (Qiagen). The pathways with *P* < 0.05 were defined as significantly perturbed by PM exposure.

### Cellular communication analysis

To identify the significant ligand-receptors in response to PM exposure in murine species, we performed homologous gene transformation based on the murine genes dataset GRCm39 downloaded from BioMart and the multicellular ligand-receptor network illustrated by Ramilowski [51]. With the transformed dataset, we applied R package iTALK to analyze the significantly shifted ligand-receptor pairs based on significant DEGs between control and exposure group. The ligand-receptor pairs we identified were categorized into 4 aspects, including “growth factor”, “cytokine”, “checkpoint” and “other”. Top 20 significant ligand-receptor pairs in each aspect were visualized by using LRPlot function of iTALK.

### Cell sub-clustering and pseudotime trajectory analysis

Sub-clustering data of neutrophils, monocytes and fibroblast was extracted from the integrated Seurat object using subset function. nPCs = 10 and resolution = 0.2, 0.15, 0.2 were set for neutrophils, monocytes and fibroblasts, respectively to better separate the subclusters. Similar processes for visualization, conserved markers identification, actual number and proportion calculation, and feature plots were performed. Pseudotime trajectory analysis was conducted by R package monocle (version 2.9). The finalized results were visualized by cell trajectory plots colored by cell states, Seurat clusters, and pseudotime scores using the plot_cell_trajectory function. Feature plots and violin plots were used to better recognize the shifted markers corresponding to immune function regulation in control and exposure groups.

### Flow cytometry analysis and sorting in bone marrow and lungs

For flow cytometry analysis, the cell suspensions derived from bone marrow, lungs and spleens were prepared and incubated with the specific antibodies cocktail at 4 °C for 30 min protected from light. In brief, the bone marrow tissues from two sides of the femur were flushed-out by ice-cold PBS with 2% FBS and the spleen tissues were grounded thoroughly by the end of syringe. The harvested cell suspensions were filtered through 70 μm cell strainers (Falcon, BD), subjected to red blood cell lysis buffer, and washed twice by PBS with 2% FBS for the following antibodies incubation. After gating the proper physical size and singlet, the antibodies used for MDSCs identification in bone marrow, spleen and lung tissues were listed below: Fixable Viability Dye eFluor™ 780 (FVD eFluor™ 780, eBioscience), anti-CD45 (clone 30-F11, PE/Cyanine5; Biolegend), anti-CD11c (clone N418, FITC; Biolegend), anti-CD11b (clone M1/70, APC; Biolegend), anti-Gr-1 (clone RB6-8C5, PE; Biolegend), anti-Ly6C (clone HK1.4, PE; Biolegend) and anti-Ly6G (clone 1A8, PE/Cyanine7; Biolegend). To identify T cells and the subsets in lung and spleen tissues, the antibodies was used as follows: FVD eFluor™ 780, anti-CD3 (clone 17A2, APC; Biolegend), anti-CD4 (clone GK1.5, FITC; Biolegend), anti-CD25 (clone 3C7, PE; Biolegend) and anti-IL-17 (TC11-18H10.1, Brilliant Violet 421; Biolegend). To distinguish macrophages from other immune cells in lung tissues, the antibodies was listed as follow: FVD eFluor™ 780 (eBioscience), anti-F4/80 (clone BM8, FITC; Biolegend), anti-CD11b (clone M1/70, PE/Cyanine5; Biolegend), anti-CD11c (clone N418, PE; Biolegend), and anti-CD206 (clone C068C2, APC; Biolegend) [52]. Flow cytometry analysis was performed using Cytoflex (Beckman Coulter), while sorting was performed using FACS Aria III (BD Biosciences). Data were analyzed using CytExpert software (Beckman Coulter) and FlowJo v10.

### Functional assays for MDSCs

MDSCs from bone marrow and lung tissues and T cells from healthy mice’s spleens were isolated as described above. Round-bottomed 96-well plates were coated with 5 μg/mL anti-CD3e monoclonal antibody (clone 145-2C11, functional grade; eBioscience) overnight at 4 °C. T cells were stained by 5 μM CellTrace™ CFSE (Invitrogen) at 37 °C for 30 min. Then, T cells were resuspended in complete culture medium (DMEM supplemented with 10% FBS, 1% P/S, 1 × antibiotic–antimycotic (Gibco), 1 × 2-mercaptoethanol (Gibco) and 2 μg/mL anti-CD28 soluble antibody (clone 37.51, functional grade; eBioscience) and seeded into the 96-well plates at a density of 1 × 10^5^ cells/well. Cells were cocultured with MDSCs at the ratio of 1:1 and 1:2 (MDSCs: T cells) for 72 h at 37 °C, 5% CO_2_. At the end of the coculturing, the cells were collected and stained with anti-CD3 antibody and then proceeded by Cytoflex. The discrete peaks of CFSE shown in the histograms made by CytExpert represent successive generations of viable T cells. Unstimulated T cells and Unstained T cells were used as two kinds of negative control to define the far-right side and far left side of the histogram. We compared the proliferation rate of T cells (%) to define the immunosuppressive function MDSCs gained under different exposure conditions. The supernatant was collected and stored at -80 °C for cytokines measurement.

### Cytokine analysis

Cytokines in lung tissues and supernatant of coculture including IL-17A and TGF-β were examined by enzyme-linked immunosorbent assay (ELISA) (Neobioscience).

### Quantitative real-time qPCR

Total RNA was extracted from the homogenized lung tissue and harvested cells by using TRIzol following manufacturer’s instructions (Thermo Fisher). The reverse transcription was conducted with the Advantage RT-for-PCR Kit (Takara), and cDNA products were used for the quantitative real-time PCR (qPCR) with SYBR Green PCR Master Mix (Toyobo). Samples were normalized to mRNA expression of internal control α-Tubulin and the results were calculated by using 2^−ΔΔCt^ method. The primers used for qPCR are listed in Additional file 1: Table S13.

### Statistical analysis

ScRNA-seq data was analyzed by R (version 3.6.3) and figures were generated with GraphPad Prism (version 7). The Venn plots were plotted by Venny (version 2.1, BioinfoBP). Statistical analysis was performed with SPSS 22.0 statistical software (SPSS Inc.). For the data analysis of ALI scores, collagen content, Ashcroft scores, pulmonary hydroxyproline content, relative mRNA expression and proportions of MDSCs, two-way analysis of variance (ANOVA) followed by Turkey’s post hoc test was used for examining the effects of PM exposure and time effect relationship. The immunosuppressive activity of MDSCs following 16-week PM exposure between IL-17A deficiency mice and their corresponding WT littermates was analyzed by two-way analysis of variance (ANOVA) followed by Turkey’s post hoc test to examine relationship between PM exposure and IL-17A presence. All results were presented as mean ± SD. Significant differences were considered at *P* < 0.05.

## Supplementary Information

**Additional file 1**. Supplementary Figures and Supplementary Tables.

**Additional file 2**. The significant differentially expressed genes (DEGs) identified for each cell cluster using FindMarkers function (Tables 1–13).

**Additional file 3**. Pathway enrichment analysis performed based on the generated DEGs of cell cluster or cell type utilizing Gene Ontology (GO) (Tables 1–13).

**Additional file 4**. Pathway enrichment analysis performed based on the generated DEGs of cell cluster or cell type utilizing Kyoto encyclopedia of genes and genomes pathway (KEGG) (Tables 1–13).

**Additional file 5**. The significantly shifted ligand-receptor pairs between control and exposure groups. The ligand-receptor pairs identified were divided into 4 categories, including “growth factor”, “cytokine”, “checkpoint” and “other” (Table 1).

**Additional file 6**. The uncropped and full-length gel and blots for Fig. S6A, C. (A) The full-length gel for Fig. S6A. (B) The uncropped blots for Fig. S6C.

## Data Availability

The datasets used and/or analyzed during the current study are available from the corresponding author on reasonable request.

## Abbreviations

PM2.5: Fine particulate matter
PM: Particulate matter
COPD: Chronic obstructive pulmonary disease
TGF-β: Transforming growth factor-β
EMT: Epithelial-mesenchymal-transition
scRNA-seq: Single-cell RNA sequencing
MDSCs: Myeloid-derived suppressor cells
ALI: Acute lung injury
CCA: Canonical correlation analysis
UMAP: Uniform Manifold Approximation and Projection
scMCA: Single-cell Mouse Cell Atlas
AMs: Alveolar macrophages
AT2: Type 2 alveolar epithelial cells
AT1: Type 1 alveolar epithelial cells
ECM: Extracellular matrix
GOBP: GO Biological Process
KEGG: Kyoto encyclopedia of genes and genomes
WT: Wild type
DEGs: Differentially expressed genes
BALF: Bronchoalveolar lavage fluid
CAP: Concentrated ambient particles
PFA: Paraformaldehyde
DMEM: Dulbecco's Modified Eagle Medium
FBS: Fetal bovine serum
BSA: Bovine serum aluminum
PCA: Principal components analysis
IPA: Ingenuity Pathway Analysis
ELISA: Enzyme-linked immunosorbent assay
ANOVA: Analysis of variance
