# Supplementary material for: Single-cell transcriptomics reveals immune dysregulation mediated by IL-17A in initiation of chronic lung injuries upon real-ambient particulate matter exposure
Source: Part Fibre Toxicol. 2022 Jun 23;19:42. doi: 10.1186/s12989-022-00483-w (PMC9219231; doi:10.1186/s12989-022-00483-w)
Supplement: Supplementary file 1 — Additional file 1. Supplementary Figures and Supplementary Tables. [file 12989_2022_483_MOESM1_ESM.docx]

Additional file 1

**Single-cell transcriptomics reveals immune dysregulation mediated by IL-17A in initiation of chronic lung injuries upon real-ambient particulate matter exposure**

Rui Zhang^#1^, Shen Chen^#1^, Liping Chen^#1^, Lizhu Ye^1^, Yue Jiang^1^, Hui Peng^1^, Zhanyu Guo^1^, Miao Li^1^, Xinhang Jiang^1^, Ping Guo^1^, Dianke Yu^2^, Rong Zhang^3^, Yujie Niu^3^, Yuan Zhuang^4^, Michael Aschner^5^, Yuxin Zheng^2^, Daochuan Li^*1^, Wen Chen^*1^

**Additional file figures**

**
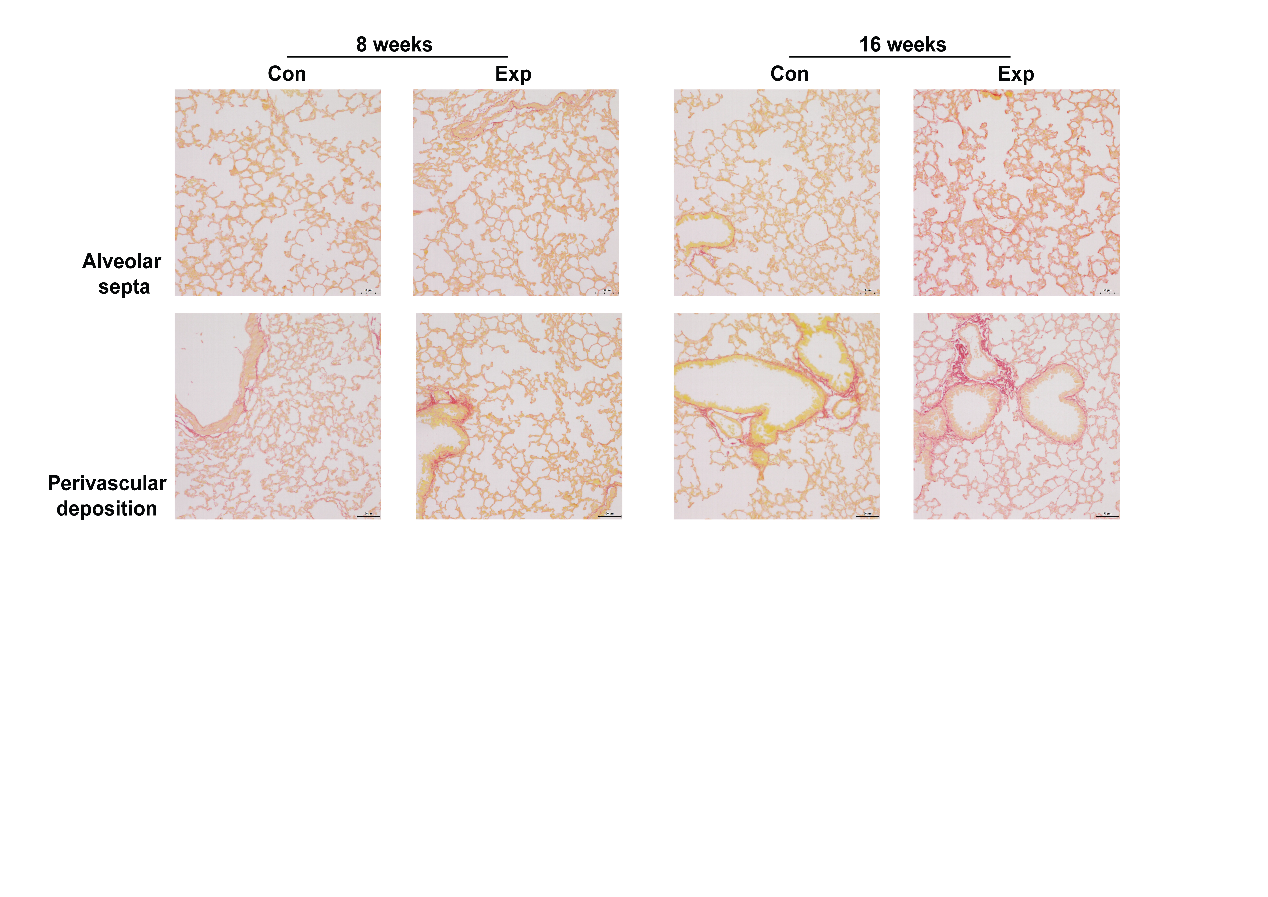
**

**Additional file 1: Fig. S1.** Effects of real-ambient PM exposure on chronic lung injury and collagen deposition. Representative images of Sirius red-stained lung sections, displaying the pathological changes in the control and exposure groups following 8-week and 16-week exposure (N = 8). Photographs were scanned by TissueFAXS analysis system. All magnifications are at 200X. Scale bar = 50 μm. The results were presented as mean ± SD. ****P*<0.001. Con: air-filtered control group; Exp: PM exposed group.


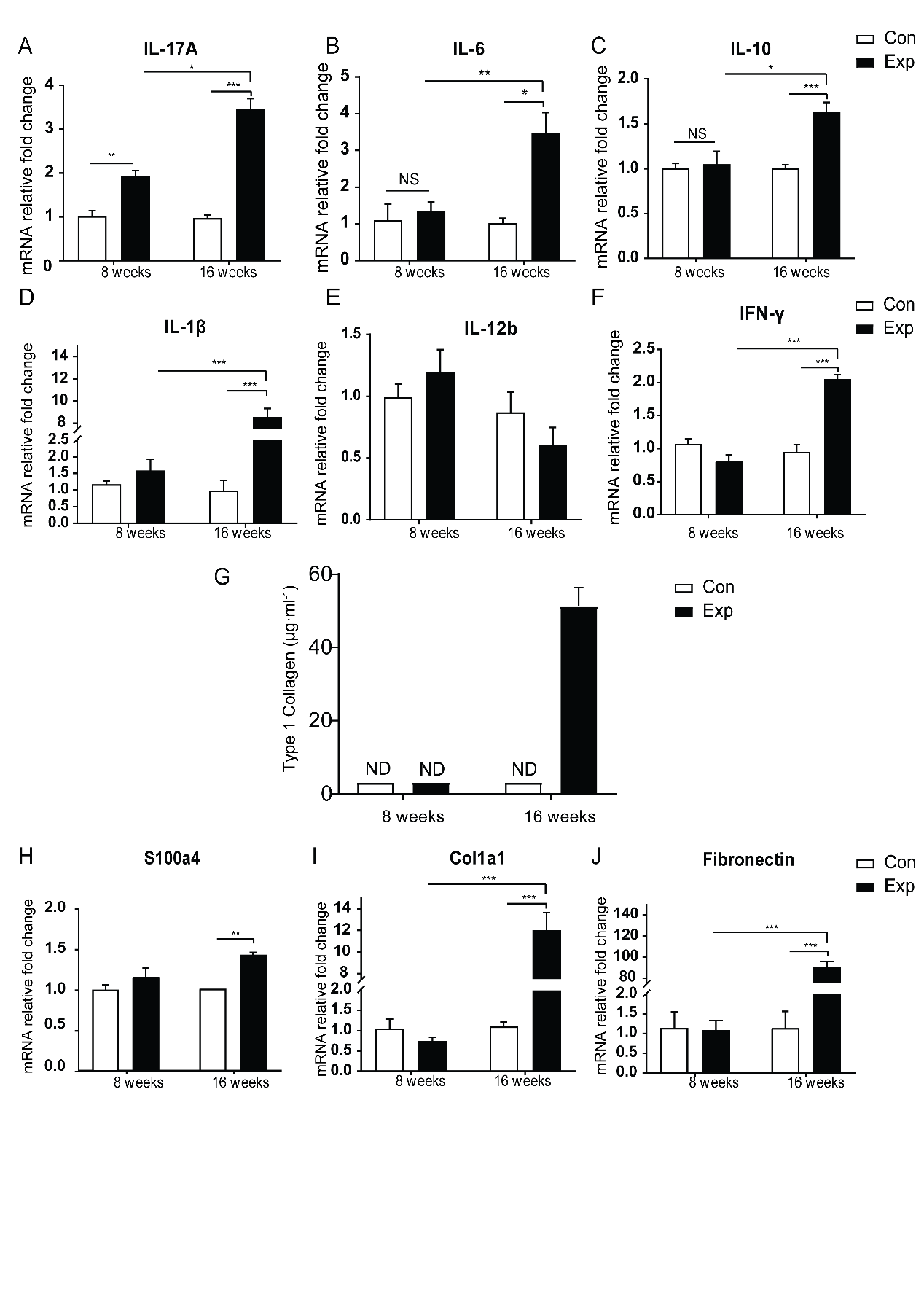


**Additional file 2: Fig. S2** Quantitative assessment of chronic lung injury and progressive pulmonary fibrosis upon sub-chronic PM exposure. (**A-F**) The relative mRNA expression levels of cytokines including IL-17A (**A)** , IL-6 (**B)**, IL-10 (**C**), IL-1β (**D**), IL-12b (**E**), and IFN-γ (**F**) in lung tissue of different groups (N = 5). (**G**) Pulmonary type 1 collagen content from control and PM-exposed mice (N = 5). (**H-J**) The relative mRNA expression levels of profibrotic factors S100a4 (**H**), Col1a1 (**I**), and Fibronectin (**J**) in lung tissue of different groups (N = 5). The results were presented as mean ± SD. ** *P*<0.01; ****P*<0.001. Con: air-filtered control group; Exp: PM exposed group; ND: not detected.

**
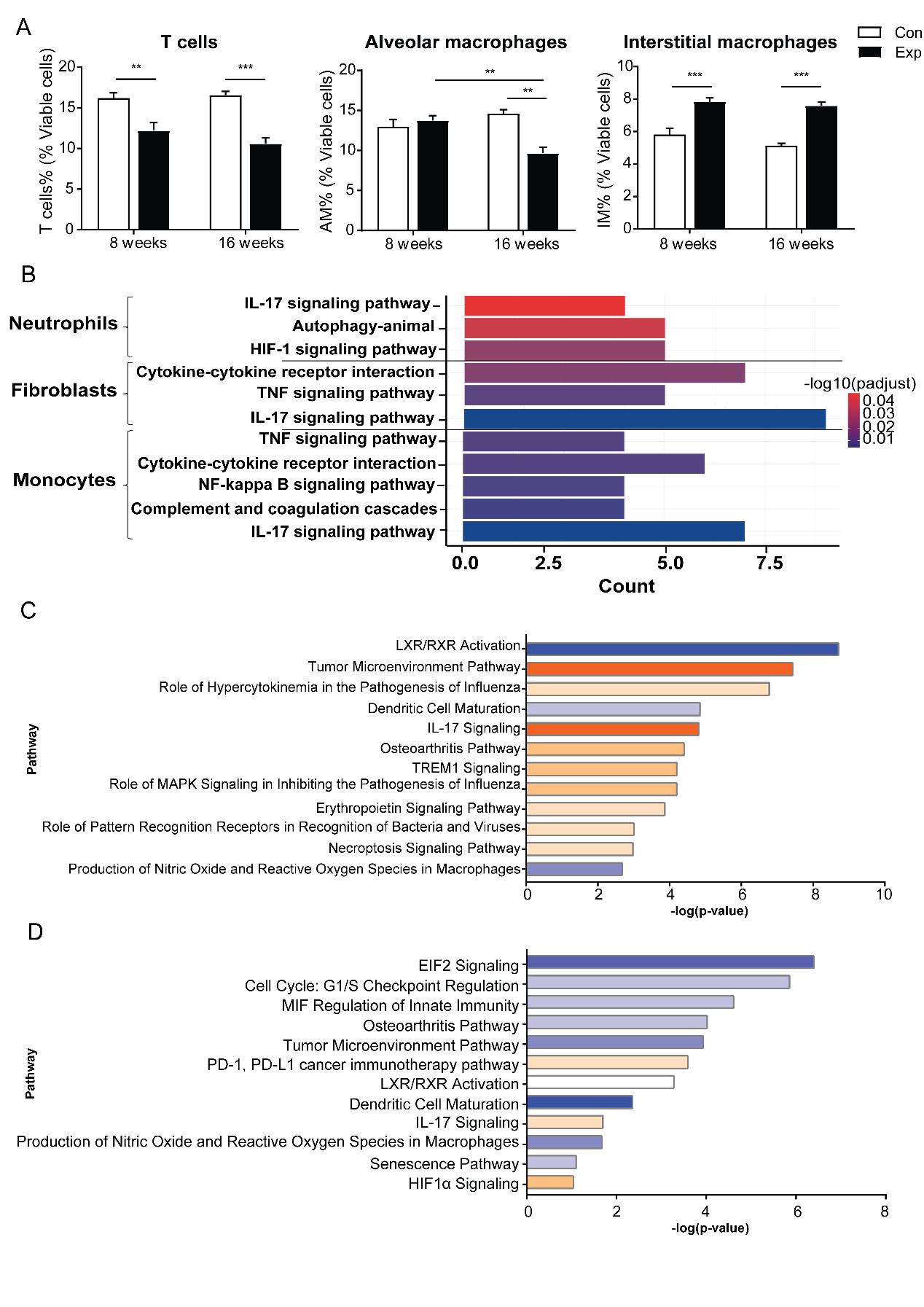
 Additional file 1: Fig. S3** Validation of scRNA-seq analysis and pathway enrichment of DEGs. (**A**) The proportions of T cells, alveolar macrophages, and interstitial macrophages detected in the viable cells of lung tissues in control and PM exposed mice (N = 4). (**B**) Top 3-5 enriched KEGG pathways among the respective DEGs in neutrophils, monocytes, and fibroblasts. (**C and D**) Key enriched canonical pathways analyzed by IPA software among the respective DEGs in monocytes (**C**) and neutrophils (**D**). Con: control group; Exp: PM exposure group.


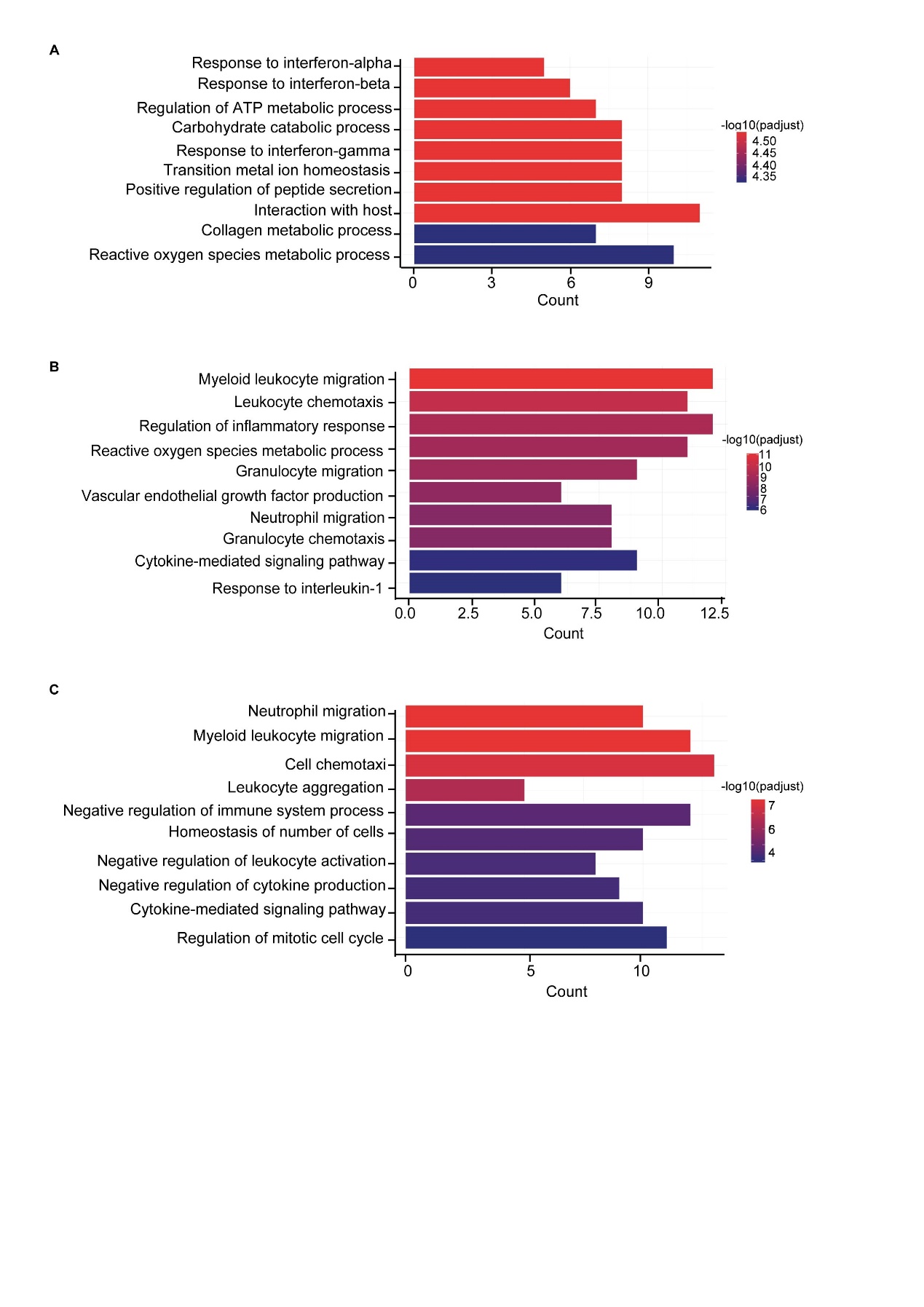


**Additional file 1: Fig. S4** Enriched GOBP programs identified among the respective DEGs in neutrophils, monocytes, and fibroblasts. (**A-C**) Enrichment analysis was carried out by GOBP programs on DEGs in neutrophils (**A**), monocytes (**B**), and fibroblasts (**C**).


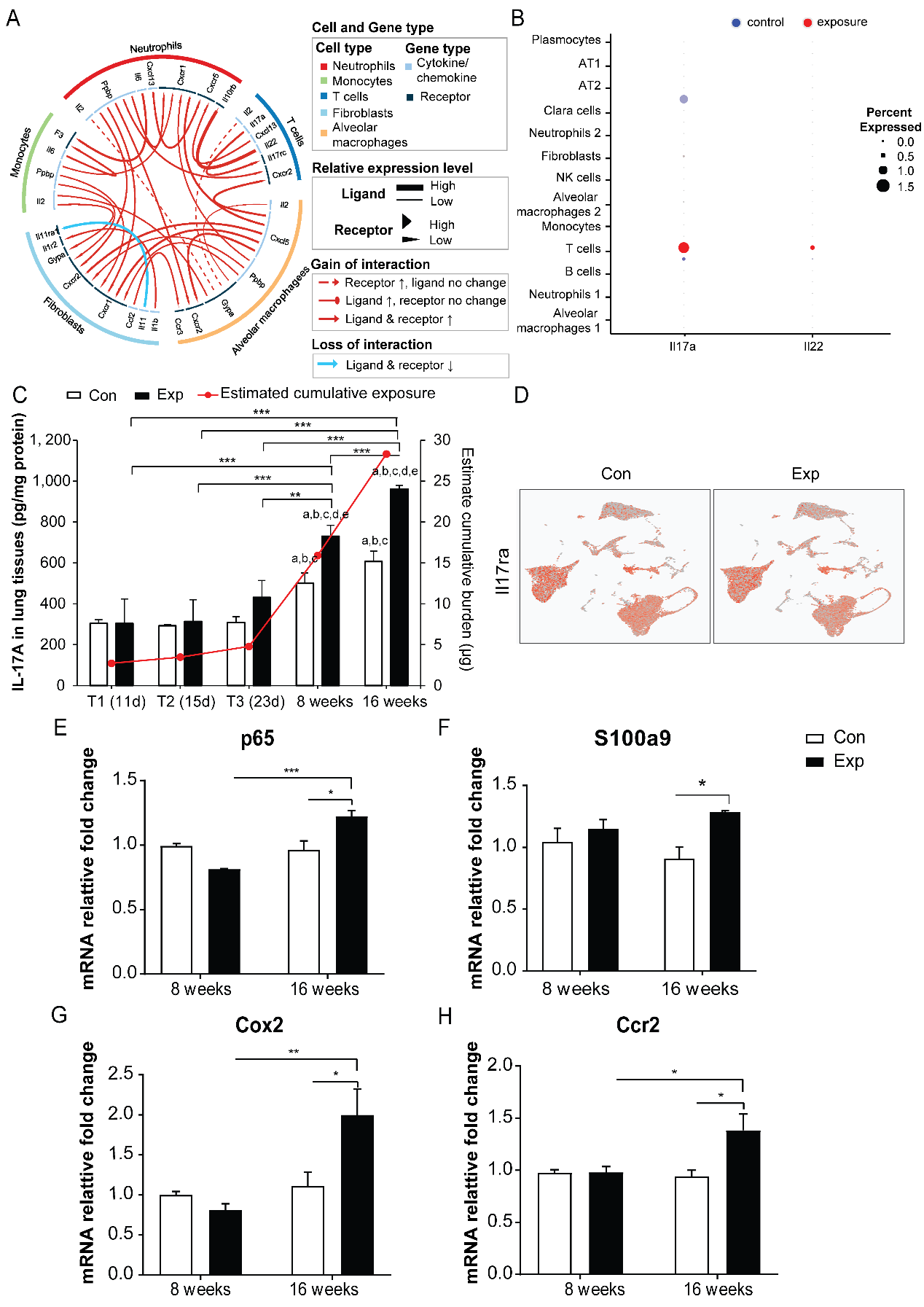


**Additional file 1: Fig. S5** IL-17 signaling pathway activation was associated with pulmonary fibrosis induced by PM exposure. (**A**) Circos plot is depicted for significant alterations (PM exposure group vs control group) in cellular interactions in cytokines among neutrophils, monocyte-derived cells (displayed as monocytes), fibroblasts, alveolar macrophages, and T cells. (**B**) The dot plot represents the expression level of the Il17a and Il22 (dot color shades) and the percentage of cells expressing two genes mentioned above (dot size). (**C**) IL-17A secretion expression in lung tissue from control and PM exposed groups at T1 (11 d), T2 (15 d), T3 (23 d), 8-weeks (56 d) and 16-weeks (112 d), and the respective cumulative exposure burdens (N = 3). The data are expressed as mean ± SD. ***P*<0.01; ****P*<0.001 compared with PM-exposed mice at different time points. ^a^*P*<0.05 compared with control mice at T1; ^b^*P*<0.05 compared with control mice at T2; ^c^*P*<0.05 compared with control mice at T3; ^d^*P*<0.05 compared with control mice at 8 weeks; ^e^ *P*<0.05 compared with control mice at 16 weeks. (**D**) Expression levels of Il17ra, which is one of the specific receptors of IL-17A on UMAP plots split by groups of different exposure status. (**E-H**) The relative mRNA expression levels of IL-17A signaling downstream genes including p65, S100a9, Cox2, and Ccr2 in lung tissue of different groups (N = 5). The results are shown as mean ± SD. **P*<0.05; ***P*<0.01; ****P*<0.001. Con: control group; Exp: PM exposure group.


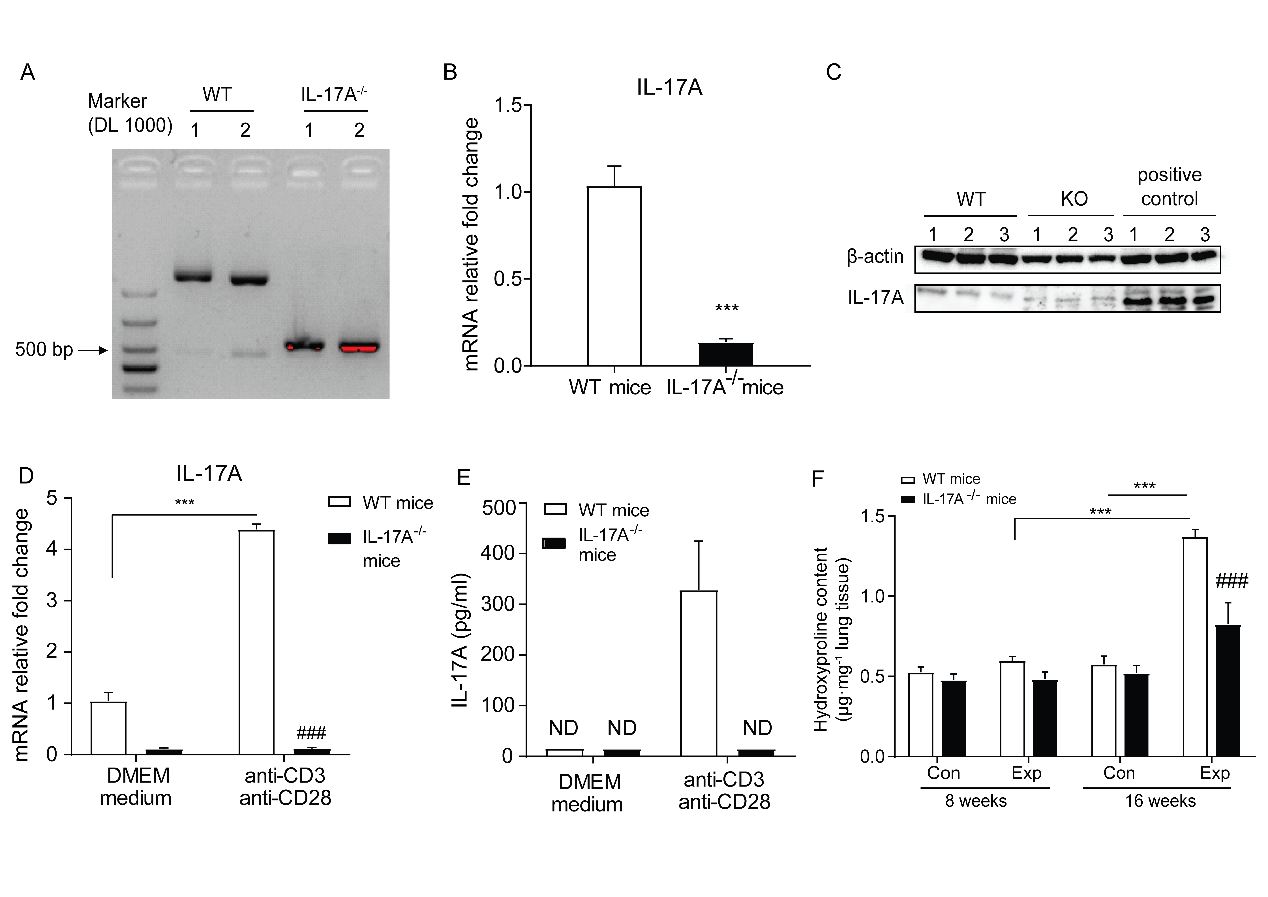


**Additional file 1: Fig. S6** Baseline information of IL-17A^-/-^ mice and the profibrotic assessment in response to PM exposure. (**A**) DNA genotyping in WT and mutant (IL-17A^-/-^) littermates by PCR analysis. The targeted bands were presented at 500 bp. (**B**) IL-17A mRNA expression in lung tissue from WT and IL-17A^-/-^ mice (N = 5). (**C**) Contents of IL-17A in lung tissue from WT and IL-17A^-/-^ mice determined by western blot. Mice intraperitoneally injected with 10 mg/kg LPS for 16 hours were used as positive control. (**D**) The relative mRNA expression levels of IL-17A in splenic T cells isolated from WT and IL-17A^-/-^ mice after stimulation with anti-CD3/anti-CD28 for 72 hours. (**E**) The secretion levels of IL-17A in the supernatant of simulated T cells from WT and IL-17A^-/-^ mice. (**F**) Pulmonary hydroxyproline content in the control and exposure groups following 8-week and 16-week exposure (N = 3). The *in vitro* experiment was carried out in 3 replicates. The results are presented as mean ± SD. *P<0.05; **P<0.01; ***P<0.001 PM-exposed mice compared with the control mice (WT--PM vs WT-Con). #P<0.05 compared with the WT PM-exposed mice (IL-17A-/--PM vs WT-PM); ###P<0.001 IL-17A-/--PM vs WT-PM. WT: wild type; Con: control group; Exp: PM exposure group; ND: not detected.


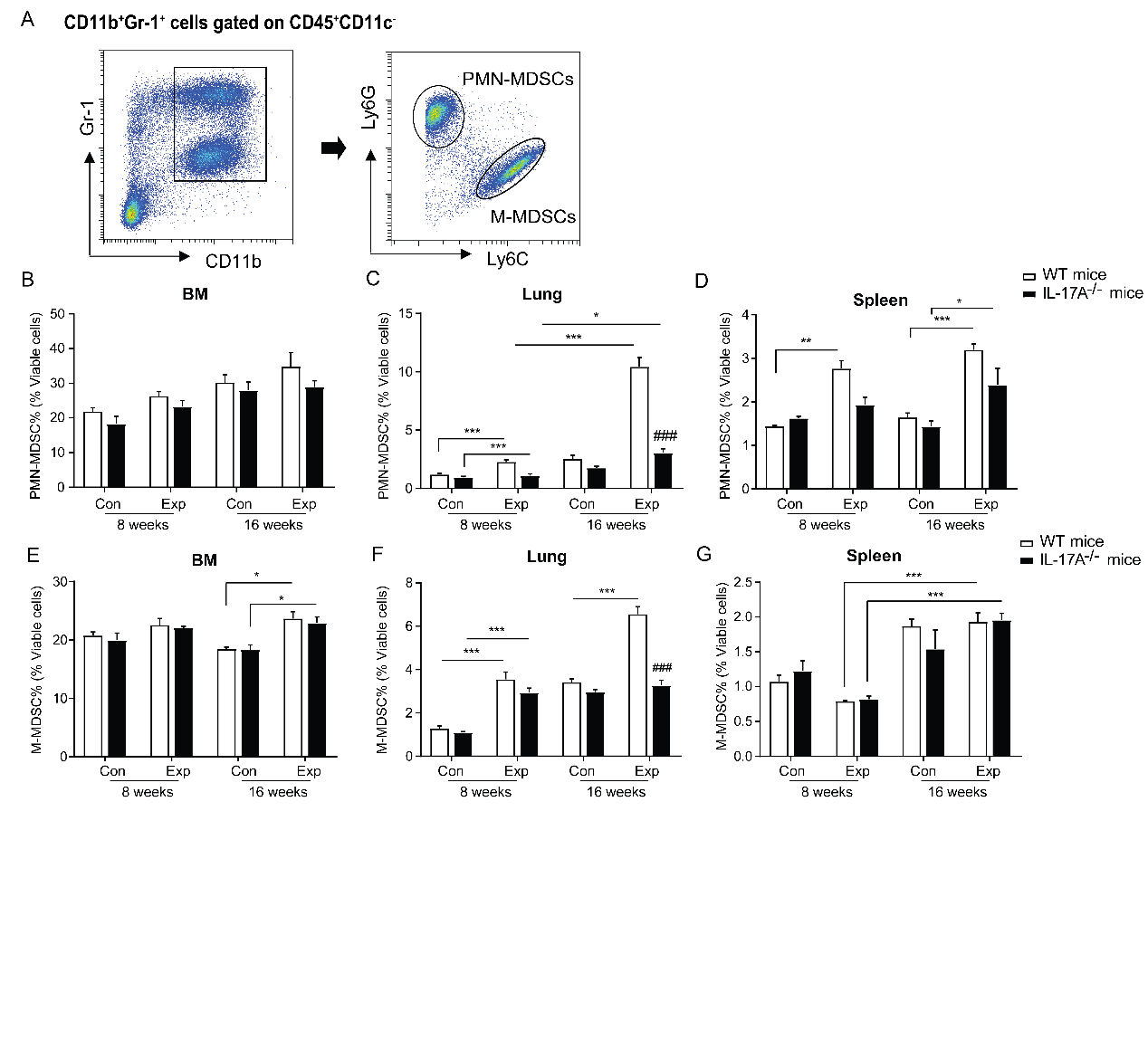


**Additional file 1: Fig. S7** Two subsets of MDSCs in multiple organs following sub-chronic exposure to PM. (**A**) Gating strategy of the flow cytometry for PMN-MDSCs (CD11b^+^Ly6G^+^Ly6C^mid^ cells) and M-MDSCs detection (CD11b^+^Ly6G^-^Ly6C^high^ cells). (**B-D**) The proportions of PMN-MDSCs detected in the viable cells of bone marrows (**B**), lung tissues (**C**), and spleens (**D**) in control and PM exposed groups from WT and IL-17A^-/-^ mice (N = 3). (**E-G**) The proportions of M-MDSCs detected in the viable cells of bone marrows (**E**), lung tissues (**F**) and spleens (**G**) in control and PM exposed groups from WT and IL-17A^-/-^ mice (N = 3). The results are presented as mean ± SD. * *P*<0.05; ** *P*<0.01; ****P*<0.001 compared with the control mice. ^###^*P*<0.001 compared with the WT PM-exposed mice (IL-17A^-/-^-PM vs WT-PM). Con: control group; Exp: PM exposure group.


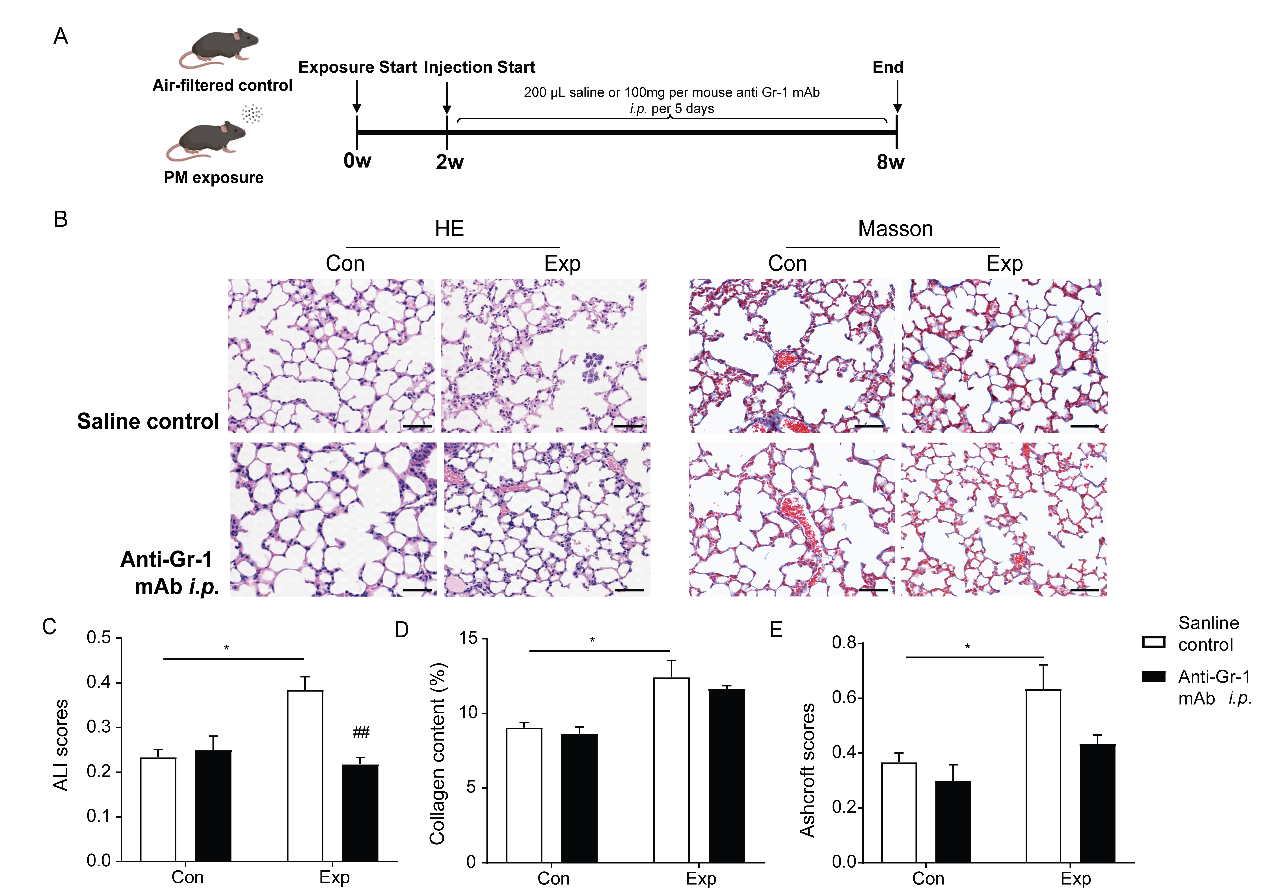


**Additional file 1: Fig. S8** MDSCs depletion leads to attenuated chronic lung injuries. (**A**) The diagram of the experimental design of MDSCs depletion following PM exposure in Shijiazhuang, China, 2019-2020. 10 mice in each group were used for anti-Gr-1 neutralizing antibody treatment or saline treatment (N = 5) through intraperitoneal injection every 5 days starting at week 2 and lasting until the end of exposure. The arrows indicate the time points of 2 weeks (2w) and 8 weeks (8w), at which anti-Gr-1 neutralizing antibody was intraperitoneally injected and the mice were sacrificed, respectively. (**B**) Representative images of H&E- and Masson's trichrome-stained lung sections, displaying the pathological changes and collagen deposition in the control and exposure groups following 8-week exposure. (**C**) ALI scores were calculated in different groups (N = 3). (**D**) Collagen content (%) in lung tissue from WT and MDSCs-depleted mice examined quantitatively on the blue area (%) of the histopathological images (N = 3). (**E**) Ashcroft scores were calculated in the groups (N = 3). The results are presented as mean ± SD. ****P*<0.001 compared with the control mice. ^###^*P*<0.001 compared with the WT PM-exposed mice (anti-Gr-1-PM vs WT-PM). Con: control group; Exp: PM exposure group.


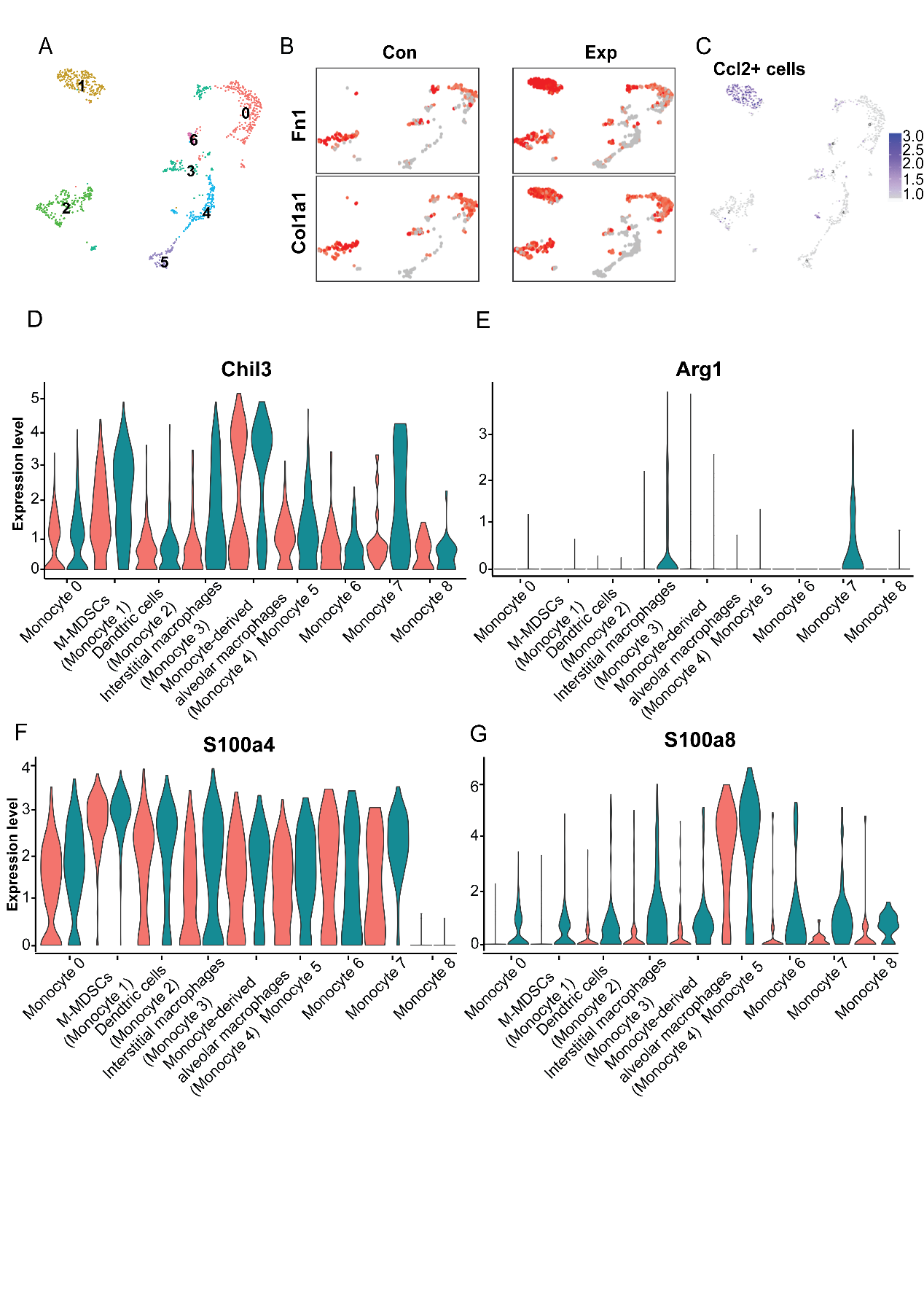


**Additional file 1: Fig. S9** Characterization of activated fibroblasts and macrophages derived from monocytes following PM exposure. (**A**) Sub-clusters identified in fibroblast subset in UMAP plot. (**B**) The expression of Fn1 and Col1a1 in fibroblast subset as shown in UMAP plots, colored from grey to red, indicating from low to high expression. Cells from control and PM exposed groups were displayed in a separate column. (**C**) Ccl2 expression in the fibroblast subset as shown in UMAP plot, color changes indicating the lowest (grey) to highest (blue) expression. (**D-G**) Violin plots display M2 profibrotic markers of monocyte-derived cell subset including Chil3 (**D**), Arg1 (**E**), S100a4 (**F**), and S100a8 (**G**) split by groups of different exposure status. Cells from control or exposed group are shown in orange or turquoise color. UMAP: Uniform Manifold Approximation and Projection; Con: control group; Exp: PM exposure group.

**Additional file tables**

**Additional file 1: Table S1. The mean PM2.5 concentration and the cumulative burden over two exposure time periods and three time points.**

| Experimental periods | Mean daily PM2.5 concentration (μg/m^3^) | | Estimate cumulative burden^a^ (μg/mouse) | PM2.5>10 μg/m^3^ (Days) | 35<PM2.5≤150 μg/m^3^ (Days) | PM2.5>150 μg/m^3^ (Days) |
| --- | --- | --- | --- | --- | --- | --- |
|  | Chamber | Ambient |  |  |  |  |
| T1 | 72.714 | 124.381 | 2.734 | 13 | 9 | 1 |
| T2 | 67.823 | 124.238 | 3.478 | 16 | 11 | 1 |
| T3 | 60.889 | 116.952 | 4.788 | 23 | 16 | 2 |
| Week 1-8 | 82.981 | 139.720 | 15.887 | 55 | 39 | 6 |
| Week 1-16 | 73.937 | 123.524 | 28.310 | 90 | 62 | 8 |

Note: a. Estimate cumulative burden=MV×T×CON×DF. MV: minute ventilation (mL/min); T: total exposure time (min); CON: mean concentration (mg/m^3^); DF: pulmonary deposition fraction (m^3^).

**Additional file 1: Table S2.** **The contents of 16 signature PAHs (ng/m^3^) on the US EPA priority pollutant list in the organic fractions of PM2.5.**

| **Parameters** | **Abbreviation** | **^a^TEF** | **Week 1-8** | **Week 1-16** |
| --- | --- | --- | --- | --- |
| Naphthalene | Nap | 0.001 | 0.14 | 0.32 |
| Acenaphthylene | AcPy | 0.001 | 0.14 | 0.15 |
| Acenaphthene | Acp | 0.001 | 0.88 | 1.12 |
| Fluorene | Flu | 0.001 | 0.60 | 0.84 |
| Phenanthrene | PA | 0.001 | 3.68 | 4.82 |
| Anthracene | Ant | 0.01 | 1.68 | 1.56 |
| Fluoranthene | FL | 0.001 | 9.61 | 12.88 |
| Pyrene | Pyr | 0.001 | 10.34 | 12.60 |
| Benzo[a]anthracene | BaA | 0.1 | 2.85 | 4.36 |
| Chrysene | Chr | 0.01 | 6.33 | 7.67 |
| Benzo[b]fluoranthene | BbF | 0.1 | 12.57 | 15.39 |
| Benzo[k]fluoranthene | BkF | 0.1 | 3.95 | 4.08 |
| Benzo[a]pyrene | BaP | 1 | 6.41 | 8.43 |
| Indeno[1,2,3-cd]pyrene | IND | 0.1 | 6.04 | 7.15 |
| Dibenzo[a,h]anthracene | DBahA | 1 | 2.19 | 2.66 |
| Benzo[g,h,i]perylene | BghiP | 0.01 | 3.58 | 3.55 |
| ∑16PAHs |  |  | 71.01 | 87.58 |
| BaPeq |  |  | 11.29 | 14.35 |

Note: ^a^ TEF, the toxic equivalency factor.

**Additional file 1: Table S3. The analysis of 18 nitro-PAHs (ng/m^3^) in the organic fractions of PM2.5.**

| **Parameters** | **Abbreviation** | **^a^TEF** | **Week 1-8** | **Week 1-16** |
| --- | --- | --- | --- | --- |
| 1-nitronaphthalene | 1-NNap |  | 0.10 | 0.15 |
| 2-nitronaphthalene | 2-NAcPy |  | 0.12 | 0.13 |
| 2-nitrobiphenyl | 2-NBP |  | 0.01 | 0.01 |
| 3-nitrobiphenyl | 3-NBP |  | 0.42 | 0.57 |
| 4-nitrobiphenyl | 4-NBP |  | 0.18 | 0.20 |
| 1,3-dinitronaphthalene | 1,3-DNNap |  | 0.01 | 0.02 |
| 3-nitrodibenzofuran | 3-NDBF |  | 0.06 | 0.13 |
| 2-nitrofluoranthene | 2-NFlu | 0.01 | 0.00 | 0.01 |
| 9-nitroanthracene | 9-NAnt | 0.0032 | 5.25 | 6.86 |
| 1,8-Dinitronaphthalene | 1,8-DNNap |  | 0.00 | 0.03 |
| 9-nitrophenanthrene | 9-NPhe |  | 0.21 | 0.75 |
| 3-nitrophenanthrene | 3-NPhe |  | 1.07 | 1.54 |
| 2-nitroanthracene | 2-NAnt |  | 0.12 | 0.14 |
| 2-fluoranthene | 2-NFL |  | 2.08 | 2.01 |
| 1-nitropyrene | 1-NPyr | 0.1 | 0.38 | 0.68 |
| 2-nitropyrene | 2-NPyr |  | 2.62 | 3.21 |
| 7-nitrobenzo[a]anthracene | 7-NBaA |  | 1.36 | 1.48 |
| 6-nitrochrysene | 6-NC | 10 | 0.28 | 0.57 |
| ∑nito-PAHs |  |  | 14.28 | 18.49 |
| TEQ |  |  | 2.88 | 5.73 |

Note: ^a^ TEF, the toxic equivalency factor.

**Additional file 1: Table S4. The analysis of 17 PCDD/F (pg/m^3^) in the organic fraction of PM2.5.**

| **Parameters** | **^a^I-TEF** | **Week 1-8** | **Week 1-16** |
| --- | --- | --- | --- |
| 2,3,7,8-TCDF | 0.1 | 0.15 | 0.21 |
| 1,2,3,7,8-PeCDF | 0.05 | 0.18 | 0.22 |
| 2,3,4,7,8-PeCDF | 0.5 | 0.25 | 0.35 |
| 1,2,3,4,7,8-HxCDF | 0.1 | 0.37 | 0.53 |
| 1,2,3,6,7,8-HxCDF | 0.1 | 0.14 | 0.24 |
| 2,3,4,6,7,8-HxCDF | 0.1 | 0.49 | 0.58 |
| 1,2,3,7,8,9-HxCDF | 0.1 | 0.13 | 0.16 |
| 1,2,3,4,6,7,8-HpCDF | 0.01 | 0.44 | 0.78 |
| 1,2,3,4,7,8,9-HpCDF | 0.01 | 0.14 | 0.17 |
| OCDF | 0.001 | 0.76 | 1.05 |
| 2,3,7,8-TCDD | 1 | 0.03 | 0.04 |
| 1,2,3,7,8-PeCDD | 0.5 | 0.05 | 0.06 |
| 1,2,3,4,7,8-HxCDD | 0.1 | 0.07 | 0.06 |
| 1,2,3,6,7,8-HxCDD | 0.1 | 0.08 | 0.07 |
| 1,2,3,7,8,9-HxCDD | 0.1 | 0.03 | 0.05 |
| 1,2,3,4,6,7,8-HpCDD | 0.01 | 0.14 | 0.28 |
| OCDD | 0.001 | 0.38 | 0.39 |
| ∑PCDF |  | 3.05 | 4.29 |
| TEQs _PCDF_ |  | 0.27 | 0.37 |
| ∑PCDD |  | 0.78 | 0.95 |
| TEQs _PCDD_ |  | 0.07 | 0.09 |
| ∑PCDF+PCDD |  | 3.83 | 5.24 |
| TEQs _PCDF+PCDD_ |  | 0.34 | 0.46 |

Note: ^a^ I-TEF, International Toxicity Equivalency Factor (I-TEF) for PCDD/F.

**Additional file 1: Table S5.** **The analysis of 18 PCB (pg/m^3^) in the organic fraction of PM2.5.**

| **Parameters** | **TEF^a^** | **Week 1-8** | **Week 1-16** |
| --- | --- | --- | --- |
| PCB 77 | 0.0001 | 0.23 | 0.22 |
| PCB 81 | 0.0003 | 0.13 | 0.11 |
| PCB 105 | 0.00003 | 0.15 | 0.23 |
| PCB 114 | 0.00003 | 0.04 | 0.09 |
| PCB 118 | 0.00003 | 0.14 | 0.16 |
| PCB 123 | 0.00003 | 0.05 | 0.03 |
| PCB 126 | 0.1 | 0.14 | 0.22 |
| PCB 156 | 0.00003 | 0.06 | 0.09 |
| PCB 157 | 0.00003 | 0.04 | 0.09 |
| PCB 167 | 0.00003 | 0.14 | 0.20 |
| PCB 169 | 0.03 | 0.03 | 0.09 |
| PCB 189 | 0.00003 | 0.14 | 0.17 |
| PCB 28 |  | 0.12 | 0.19 |
| PCB 52 |  | 0.14 | 0.17 |
| PCB 101 |  | 0.29 | 0.42 |
| PCB 138 |  | 0.13 | 0.19 |
| PCB 153 |  | 0.22 | 0.18 |
| PCB 180 |  | 0.13 | 0.23 |
| 𝛴 PCB |  | 2.32 | 3.08 |
| TEQs PCB |  | 0.02 | 0.02 |

Note: ^a^ TEF, the 2005 World Health Organization reevaluation of human and mammalian toxic equivalency factors (TEFWHO-05) for PCB.

**Additional file 1: Table S6.** **The analysis of elements and ion species (ng/m^3^) in water-soluble components of PM2.5.**

| **Parameters** | **Week 1-8** | **Week 1-16** |
| --- | --- | --- |
| **Metal element** |  |  |
| Li | 0.21 | 0.28 |
| Be | BDL | BDL |
| Na | 394.63 | 372.25 |
| Mg | 22.86 | 30.74 |
| Al | 9.52 | 8.58 |
| K | 202.20 | 202.16 |
| Ca | 271.96 | 397.17 |
| Ti | 0.59 | 0.35 |
| V | 0.06 | 0.09 |
| Cr | 0.09 | 0.12 |
| Mn | 0.88 | 1.25 |
| Fe | 0.77 | 1.30 |
| Co | 0.00 | 0.01 |
| Ni | 0.03 | 0.05 |
| Cu | 0.45 | 0.64 |
| Zn | 30.89 | 29.39 |
| Ga | 0.06 | 0.17 |
| As | 1.67 | 1.18 |
| Se | 0.19 | 0.33 |
| Rb | 0.38 | 0.32 |
| Sr | 1.78 | 1.38 |
| Y | BDL | BDL |
| Zr | 0.04 | 0.04 |
| Nb | 0.00 | 0.00 |
| Mo | 0.05 | 0.16 |
| Pd | 0.00 | 0.00 |
| Ag | BDL | BDL |
| Cd | 0.05 | 0.08 |
| Sn | 0.05 | 0.06 |
| Sb | 0.06 | 0.09 |
| Te | 0.00 | 0.00 |
| Ba | 1.43 | 2.27 |
| Ta | BDL | BDL |
| W | 0.04 | 0.08 |
| Tl | 0.04 | 0.03 |
| Pb | 0.17 | 0.25 |
| 𝛴 Metal | 941.16 | 1050.80 |
| **Anion** |  |  |
| F^-^ | 39.78 | 35.30 |
| Cl^-^ | 555.95 | 451.29 |
| SO_4_^2-^ | 1782.89 | 2344.53 |
| NO_3_^-^ | 1619.87 | 2095.91 |
| PO_4_^-^ | 7.17 | 5.98 |
| NO^2-^ | 3.79 | 4.52 |
| Br^-^ | 1.78 | 2.01 |
| 𝛴 Anion | 4011.23 | 4939.55 |

Note: BDL, below detective level.

**Additional file 1: Table S7. Individual value of ALI score, collagen content (%) and Ashcroft score following 8-week and 16-week PM exposure**

| **Group** | **Sample ID** | **ALI score** | **Collagen content (%)** | **Ashcroft score** |
| --- | --- | --- | --- | --- |
| Con-8 weeks | 1 | 0.216 | 9.867 | 0.300 |
|  | 2 | 0.182 | 9.556 | 0.200 |
|  | 3 | 0.216 | 9.160 | 0.200 |
|  | 4 | 0.148 | 8.146 | 0.000 |
|  | 5 | 0.222 | 10.061 | 0.300 |
|  | 6 | 0.140 | 8.764 | 0.100 |
|  | 7 | 0.210 | 7.946 | 0.000 |
|  | 8 | 0.245 | 8.346 | 0.000 |
| Exp-8 weeks | 9 | 0.965 | 11.373 | 0.600 |
|  | 10 | 0.920 | 10.321 | 0.500 |
|  | 11 | 0.895 | 9.226 | 0.400 |
|  | 12 | 0.965 | 9.546 | 0.400 |
|  | 13 | 0.675 | 9.178 | 0.300 |
|  | 14 | 0.885 | 9.835 | 0.400 |
|  | 15 | 0.745 | 10.277 | 0.500 |
|  | 16 | 0.490 | 9.062 | 0.200 |
| Con-16 weeks | 17 | 0.210 | 10.650 | 0.300 |
|  | 18 | 0.153 | 10.790 | 0.500 |
|  | 19 | 0.233 | 8.699 | 0.000 |
|  | 20 | 0.268 | 10.650 | 0.200 |
|  | 21 | 0.150 | 8.094 | 0.000 |
|  | 22 | 0.249 | 10.962 | 0.400 |
|  | 23 | 0.255 | 9.611 | 0.100 |
|  | 24 | 0.150 | 7.662 | 0.000 |
| Exp-16 weeks | 25 | 0.512 | 19.540 | 1.500 |
|  | 26 | 0.465 | 17.350 | 1.270 |
|  | 27 | 0.430 | 21.460 | 1.100 |
|  | 28 | 0.255 | 15.100 | 1.000 |
|  | 29 | 0.484 | 16.200 | 1.000 |
|  | 30 | 0.395 | 15.187 | 1.000 |
|  | 31 | 0.397 | 17.918 | 1.100 |
|  | 32 | 0.339 | 17.580 | 1.300 |

**Additional file 1: Table S8. Detailed information and reproducibility of each sample in scRNA-seq analysis**

| **Sample ID** | **Sample Contents** | **Estimated cell counts in raw data** | **nUMIs in raw data** | **Estimated cell counts after quality control** | **nUMIs after quality control** |
| --- | --- | --- | --- | --- | --- |
| Con-1 | data from sample 1 in control group | 9471 | 31053 | 8394 | 18608 |
| Con-2 | data from sample 2 in control group | 9417 | 31053 | 8321 | 18388 |
| Con-3 | data from sample 3 in control group | 11332 | 31053 | 10039 | 17926 |
| Exp-1 | data from sample 1 in exposure group | 9209 | 31053 | 8140 | 17624 |
| Exp-2 | data from sample 2 in exposure group | 10802 | 31053 | 9503 | 18987 |
| Exp-3 | data from sample 3 in exposure group | 10832 | 31053 | 9003 | 18782 |
| Con-Aggr | data pooled from 3 samples in control group by using Cellranger aggr | 30220 | 31053 | 26745 | 20160 |
| Exp-Aggr | data pooled from 3 samples in exposure group by using Cellranger aggr | 30843 | 31053 | 26532 | 20367 |

**Additional file 1: Table S9.** **Top 10 conserved markers of 13 cell clusters used for cell type annotations in scRNA-seq dataset.**

| Seurat cluster | annotation | Top 10 conserved markers identified by FindConservedMarkers function | | | | | | | | | | |
| --- | --- | --- | --- | --- | --- | --- | --- | --- | --- | --- | --- | --- |
| 0 | Alveolar macrophages | Chil3 | Lpl | Plet1 | Ear2 | Mrc1 | Abcg1 | Atp6v0d2 | Krt79 | Ear1 | Tcf7l2 |  |
| 1 | Neutrophils | S100a9 | S100a8 | Retnlg | Il1b | Ifitm1 | Clec4d | Hdc | Cxcr2 | Slc7a11 | Csf3r |  |
| 2 | B cells | Cd79a | Ly6d | Igkc | Ebf1 | Cd79b | Ms4a1 | Cd74 | Ighm | Ighd | H2-Eb1 |  |
| 3 | T cells | Ccl5 | Il7r | Trbc2 | Ms4a4b | Cd3g | Cd3d | Trbc1 | H2-Q7 | Ms4a6b | Bcl11b |  |
| 4 | Monocytes | Apoe | S100a4 | C1qa | Plac8 | C1qb | Ms4a6c | Cst3 | Ccr2 | Crip1 | Csf1r |  |
| 5 | Alveolar macrophages | Stmn1 | Mki67 | Top2a | Tubb5 | Tuba1b | Pclaf | Cenpf | Birc5 | Cenpe | Atad2 |  |
| 6 | NK cells | Gzma | Ccl5 | AW112010 | Nkg7 | Il2rb | Prf1 | Serpinb9 | Gzmb | Klra4 | Klrd1 |  |
| 7 | Fibroblasts | Dcn | Mgp | Igfbp5 | Col1a2 | Col3a1 | Sparc | Aebp1 | Serping1 | Rarres2 | Col1a1 |  |
| 8 | Neutrophils | Ebf1 | Ms4a1 | Ly6d | Bank1 | Cd79b | Pax5 | Ralgps2 | Ighd | Iglc2 | Cd79a |  |
| 9 | Clara cells | Ppbp | Myl9 | Alox12 | Nrgn | Itga2b | Aldh1a1 | Gng11 | Wfdc2 | Sftpa1 | Pf4 |  |
| 10 | AT2 | Top2a | Hist1h1b | Hist1h2ap | Pclaf | Cenpf | Hist1h2ae | Ube2c | Hist1h1e | Birc5 | Kif11 |  |
| 11 | AT1 | Sec14l3 | Ccdc153 | Dnah5 | Tmem212 | Cyp2s1 | Dynlrb2 | Dnah12 | Dnah6 | Ces1d | Fam183b |  |
| 12 | Plasmocytes | Jchain | Iglv1 | Derl3 | Igha | Iglc1 | Mzb1 | Fam46c | Iglc2 | Iglc3 | Txndc5 |  |

**Additional file 1: Table S10. Individual value of ALI score, collagen content (%) and Ashcroft scoreof WT and IL-17A^-/-^ mice following 8-week and 16-week PM exposure**

| **Group** | **Sample ID** | **ALI score** | **Collagen content (%)** | **Ashcroft score** | **Group** | **Sample ID** | **ALI score** | **Collagen content (%)** | **Ashcroft score** |
| --- | --- | --- | --- | --- | --- | --- | --- | --- | --- |
| WT-Con 8 weeks | 1 | 0.230 | 12.594 | 0.300 | KO-Con 8 weeks | 33 | 0.240 | 10.041 | 0.200 |
|  | 2 | 0.387 | 10.816 | 0.100 |  | 34 | 0.396 | 9.999 | 0.000 |
|  | 3 | 0.361 | 11.801 | 0.300 |  | 35 | 0.375 | 11.254 | 0.400 |
|  | 4 | 0.216 | 13.810 | 0.400 |  | 36 | 0.256 | 11.704 | 0.200 |
|  | 5 | 0.291 | 10.790 | 0.000 |  | 37 | 0.268 | 11.630 | 0.000 |
|  | 6 | 0.317 | 10.650 | 0.000 |  | 38 | 0.120 | 11.667 | 0.400 |
|  | 7 | 0.222 | 11.380 | 0.000 |  | 39 | 0.226 | 9.066 | 0.000 |
|  | 8 | 0.312 | 10.650 | 0.200 |  | 40 | 0.205 | 11.015 | 0.100 |
| WT-Exp 8 weeks | 9 | 0.542 | 13.185 | 0.700 | KO-Exp 8 weeks | 41 | 0.389 | 9.207 | 0.100 |
|  | 10 | 0.606 | 11.862 | 0.400 |  | 42 | 0.268 | 9.440 | 0.300 |
|  | 11 | 0.540 | 12.963 | 0.600 |  | 43 | 0.376 | 9.324 | 0.300 |
|  | 12 | 0.484 | 12.220 | 0.500 |  | 44 | 0.336 | 9.479 | 0.500 |
|  | 13 | 0.512 | 12.805 | 0.500 |  | 45 | 0.380 | 9.818 | 0.600 |
|  | 14 | 0.457 | 10.090 | 0.400 |  | 46 | 0.270 | 11.443 | 0.400 |
|  | 15 | 0.473 | 12.903 | 0.500 |  | 47 | 0.240 | 10.565 | 0.500 |
|  | 16 | 0.428 | 12.832 | 0.500 |  | 48 | 0.237 | 10.992 | 0.500 |
| WT-Con 16 weeks | 17 | 0.326 | 9.403 | 0.500 | KO-Con 16 weeks | 49 | 0.286 | 12.486 | 0.300 |
|  | 18 | 0.229 | 12.422 | 0.200 |  | 50 | 0.272 | 10.312 | 0.100 |
|  | 19 | 0.314 | 11.637 | 0.400 |  | 51 | 0.292 | 12.722 | 0.400 |
|  | 20 | 0.340 | 14.605 | 0.200 |  | 52 | 0.334 | 9.431 | 0.000 |
|  | 21 | 0.341 | 13.850 | 0.300 |  | 53 | 0.355 | 11.098 | 0.300 |
|  | 22 | 0.233 | 11.154 | 0.100 |  | 54 | 0.231 | 13.325 | 0.300 |
|  | 23 | 0.201 | 10.957 | 0.100 |  | 55 | 0.310 | 10.720 | 0.000 |
|  | 24 | 0.254 | 10.675 | 0.000 |  | 56 | 0.219 | 11.020 | 0.000 |
| WT-Exp 16 weeks | 25 | 0.426 | 20.184 | 1.700 | KO-Exp 16 weeks | 57 | 0.355 | 11.883 | 0.600 |
|  | 26 | 0.376 | 17.261 | 1.400 |  | 58 | 0.382 | 10.337 | 0.400 |
|  | 27 | 0.428 | 17.199 | 1.300 |  | 59 | 0.334 | 12.443 | 0.600 |
|  | 28 | 0.412 | 17.115 | 0.900 |  | 60 | 0.328 | 14.622 | 0.900 |
|  | 29 | 0.438 | 16.314 | 1.100 |  | 61 | 0.348 | 11.859 | 0.700 |
|  | 30 | 0.389 | 14.550 | 0.800 |  | 62 | 0.227 | 11.711 | 0.800 |
|  | 31 | 0.206 | 14.295 | 1.000 |  | 63 | 0.219 | 10.427 | 0.700 |
|  | 32 | 0.376 | 15.777 | 1.100 |  | 64 | 0.198 | 13.291 | 0.600 |

**Additional file 1: Table S11.** **Top 10 specific markers of 9 cell clusters used for cell type annotations in monocyte subset.**

| Seurat subset cluster | | annotation | Top 10 markers identified by FindAllMarkers function | | | | | | | | | | Con | Exp |
| --- | --- | --- | --- | --- | --- | --- | --- | --- | --- | --- | --- | --- | --- | --- |
| 0 | monocyte 1 | | Ace | Cd300e | Treml4 | Spn | Adgre4 | Pou2f2 | Itgal | Gngt2 | Stap1 | Dusp16 | 557 | 483 |
| 1 | M-MDSC/immature monocyte | | Ly6c2 | Ms4a4c | Plac8 | Ccr2 | Lyz2 | F13a1 | S100a4 | Slfn5 | Ifi27l2a | Thbs1 | 330 | 471 |
| 2 | dendritic cell | | H2-Eb1 | H2-Ab1 | H2-Aa | H2-DMb1 | H2-DMa | Cd74 | Cd209a | Mgl2 | Ccl17 | Retnla | 265 | 297 |
| 3 | interistial macrophage | | Fcna | C1qc | C1qa | Pf4 | C1qb | Lyve1 | Cxcl13 | Prg4 | Ccl8 | Slpi | 156 | 341 |
| 4 | monocyte 5 | | Atp6v0d2 | Pmepa1 | Lmna | Ctsd | Cd63 | Cd9 | Plet1 | Abcg1 | Lpl | Chil3 | 181 | 129 |
| 5 | monocyte 6 | | Cxcr2 | Retnlg | Mmp9 | Hdc | S100a8 | S100a9 | Slc7a11 | G0s2 | Csf3r | Il1b | 122 | 130 |
| 6 | monocyte 7 | | Fscn1 | Ccl22 | Ccr7 | Serpinb9 | Ccl17 | Tbc1d4 | Ccl5 | Rgs1 | Basp1 | Tmem123 | 82 | 66 |
| 7 | monocyte 8 | | Cenpf | Pclaf | Birc5 | Ube2c | Top2a | Mki67 | Stmn1 | Tubb5 | Tuba1b | Hmgb2 | 17 | 53 |
| 8 | monocyte 9 | | Klk1 | Cox6a2 | Ccr9 | Siglech | Iglc3 | Spib | Ly6d | Tcf4 | Bst2 | Irf8 | 26 | 25 |

**Additional file 1: Table S12.** **Top 10 specific markers of 9 cell clusters used for cell type annotations in neutrophil subset.**

| Seurat subset cluster | annotation | Top 10 conserved markers identified by FindAllMarkers function | | | | | | | | | | Con | Exp |
| --- | --- | --- | --- | --- | --- | --- | --- | --- | --- | --- | --- | --- | --- |
| 0 | neutrophil 1 | Atf3 | Rgs2 | Vps37b | Cd300lb | D8Ertd738e | Yeats4 | Thbs1 | Eprs | Cstb | Sh2d3c | 3314 | 1481 |
| 1 | neutrophil 2 | Hbb-bs | Wfdc21 | Lcn2 | Mmp8 | Retnlg | Plin2 | Slc15a2 | Sipa1l1 | Gm5483 | Ly6g | 6 | 3719 |
| 2 | PMN-MDSC/  immature neutrophil | Scgb1a1 | Wfdc17 | Ifitm1 | Steap4 | Tspo | Prr5l | Cd244 | Ppp1r3b | Gm28192 | Cdkn1b | 22 | 3233 |
| 3 | neutrophil 4 | Nfkbia | Tnfaip3 | Nfkbiz | Marcksl1 | Gadd45b | Dusp2 | Ccrl2 | Nfkbid | Icam1 | Bcl2a1b | 1540 | 853 |
| 4 | neutrophil 5 | Ifit1 | Ifit3 | Ifit3b | Rsad2 | Slfn5 | Isg15 | Slfn4 | Ifi204 | Gbp2 | Ifi27l2a | 136 | 365 |
| 5 | neutrophil 6 | Rpl12 | Rps20 | Rps18 | Rpsa | Cd74 | Rpl10a | Rps19 | Rpl32 | Chil3 | Igkc | 43 | 70 |

**Additional file 1: Table S13.** **Primer lists for qRT-PCR.**

| **Gene** | **Forward Primer (5'-3')** | **Reverse Primer (5'-3')** |
| --- | --- | --- |
| IL1β | ATGGGCAACCACTTACCTATTT | GTTCTAGAGAGTGCTGCCTAATG |
| IL12b | GATGTGTCCTCAGAAGCTAACC | CCAGTCCACCTCTACAACATAAA |
| IL17a | CAAACATGAGTCCAGGGAGAG | GCTGAGCTTTGAGGGATGAT |
| IL6 | GTCTGTAGCTCATTCTGCTCTG | GAAGGCAACTGGATGGAAGT |
| IL10 | ACAGCCGGGAAGACAATAAC | CAGCTGGTCCTTTGTTTGAAAG |
| TGFβ1 | GGTGGTATACTGAGACACCTTG | CCCAAGGAAAGGTAGGTGATAG |
| Nos2 | TCTCCCTTTCCTCCCTTCTT | CTTCAGTCAGGAGGTTGAGTTT |
| IFNγ | GGCCATCAGCAACAACATAAG | GTTGACCTCAAACTTGGCAATAC |
| Ccl2 | CTCGGACTGTGATGCCTTAAT | TGGATCCACACCTTGCATTTA |
| Ccr2 | TTACACCTGTGGCCCTTATTT | CTGAGTAGCAGATGACCATGAC |
| S100a4 | ATTCAGCACTTCCTCTCTCTTG | CACCCTCTTTGCCTGAGTATT |
| Acta2 | CCATCATGCGTCTGGACTT | GGCAGTAGTCACGAAGGAATAG |
| Col1a1 | GCTTGAAGACCTATGTGGGTATAA | GGTGGAGAAAGGAGCAGAAA |
| Fibronectin | TCCTGTCTACCTCACAGACTAC | GTCTACTCCACCGAACAACAA |
| p65 | GGTGCATCCCTGTGTTGATA | CGTGGAGGAAGACACTTGATAG |
| S100a9 | GCAAGAAGATGGCCAACAAAG | GGTGTCCTTCCTTCCTAGAGTA |
| Cox2 | CGGACTGGATTCTATGGTGAAA | CTTGAAGTGGGTCAGGATGTAG |
| Arg1 | CAGAGGTCCAGAAGAATGGAAG | TCCACCCAAATGACACATAGG |
| Cd206 | GGAATCAAGGGCACAGAGTTA | TTCCATCTGCTCCACAATCC |
| Ym1 | GCTAAGGACAGGCCAATAGAA | GCATTCCAGCAAAGGCATAG |
| α-Tubulin | GGTGATGTGGTTCCCAAAGA | GTGGGAGGCTGGTAGTTAATG |
