# Supplementary material for: Single-cell transcriptomics reveals immune dysregulation mediated by IL-17A in initiation of chronic lung injuries upon real-ambient particulate matter exposure
Source: Part Fibre Toxicol. 2022 Jun 23;19:42. doi: 10.1186/s12989-022-00483-w (PMC9219231; doi:10.1186/s12989-022-00483-w)
Supplement: Supplementary file 6 — Additional file 6. The uncropped and full-length gel and blots for Fig. S6A, C. (A) The full-length gel for Fig. S6A. (B) The uncropped blots for Fig. S6C. [file 12989_2022_483_MOESM6_ESM.docx]

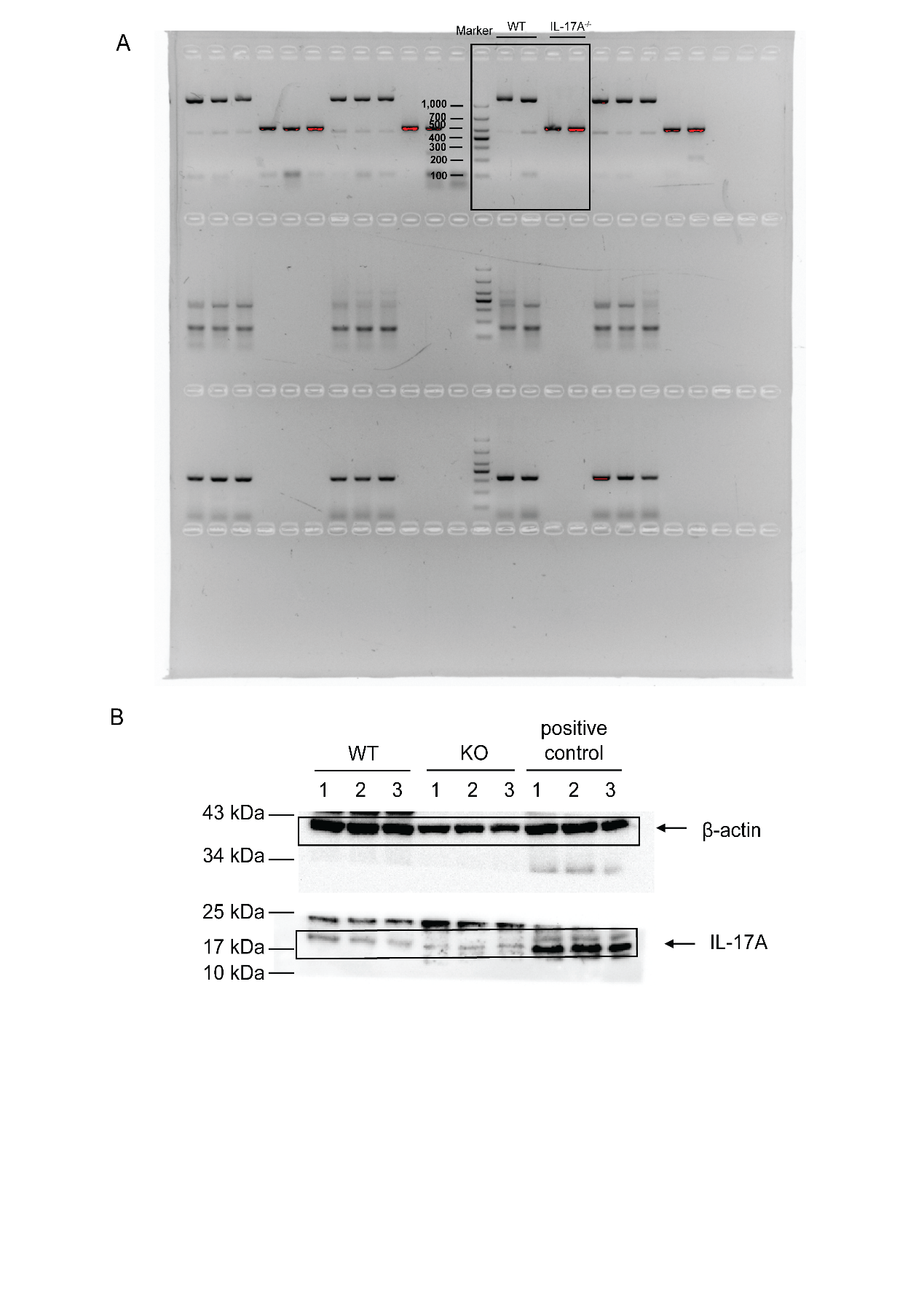


**Additional file 6.** The uncropped and full-length gel and blots for **Fig. S6A, C**. (**A**) The full-length gel for **Fig. S6A**. (**B**) The uncropped blots for **Fig. S6C**.
